# Hyperandrogenism in polycystic ovary syndrome augments Estrogen synthesis through AR-FOXL2–mediated activation of the aromatase gene in granulosa cells

**DOI:** 10.1186/s13048-025-01790-4

**Published:** 2025-09-02

**Authors:** Yi-Ru Tsai, Yen-Nung Liao, Cheng-Ju Tsai, Yu-Ang Lee, Shih-Min Hsia, Kuo-Chung Lan, Hong-Yo Kang

**Affiliations:** 1 Graduate Institute of Clinical Medical Sciences, College of Medicine, Chang Gung University, Taoyuan, 333 Taiwan; 2 An-Ten Obstetrics and Gynecology Clinic, Kaohsiung, 802 Taiwan; 3 Department of Chinese Medicine, College of Medicine, Kaohsiung Chang Gung Memorial Hospital, Chang Gung University, Kaohsiung, 833 Taiwan; 4 Graduate Institute of Metabolism and Obesity Sciences, School of Nutrition and Health Sciences, College of Nutrition, Taipei Medical University, Taipei, 110 Taiwan; 5 Center for Hormone and Reproductive Medicine Research, Department of Obstetrics and Gynecology, College of Medicine, Kaohsiung Chang Gung Memorial Hospital, Chang Gung University, Kaohsiung, 833 Taiwan; 6 Division of Endocrinology and Metabolism, Department of Internal Medicine, College of Medicine, Kaohsiung Chang Gung Memorial Hospital, Chang Gung University, Kaohsiung, 833 Taiwan; 7 Department of Biological Science, National Sun Yat-sen University, Kaohsiung, 804 Taiwan

**Keywords:** Polycystic ovary syndrome, Hyperandrogenism, AR, FOXL2, CYP19A1

## Abstract

**Background:**

Polycystic ovary syndrome (PCOS) is a complex disorder characterized by various reproductive, metabolic, and endocrine abnormalities. Hyperandrogenism is a key feature of PCOS that significantly impacts ovarian function. However, its effects on granulosa cells (GCs) function for estrogen production in PCOS remains limited.

**Methods:**

Mouse PCOS-like models were used to investigate the effects of androgen treatments on androgen receptor (AR) downstream gene expression in preantral follicles and primary GCs by qPCR, immunohistochemistry. The expression pattern of AR and FOXL2 was analyzed by single-cell RNA-sequencing analysis, immunohistochemical, and immunofluorescence staining. The AR-FOXL2 interaction was characterized using molecular docking and immunoprecipitation. The functional regulation of the *CYP19A1* promoter by AR-FOXL2 was further analyzed using chromatin immunoprecipitation and dual-luciferase reporter assay. Effects of AR knockdown and inhibitors on estrogen secretion were evaluated in cultured follicles and GCs. Clinical specimens from PCOS patients with hyperandrogenism were analyzed for estrone and estradiol levels in follicular fluid and gene expression in GCs.

**Results:**

Single-cell RNA sequencing revealed similar expression patterns of AR and FOXL2 across ovarian cell populations. Dihydrotestosterone increased AR protein expression, particularly in preantral follicles, and upregulated both AR and FOXL2 target genes. *CYP19A1* gene that encodes aromatase was significantly elevated in androgens-treated ovaries, follicles, and GCs. Immunofluorescence and co-immunoprecipitation demonstrated that androgen treatment promotes AR-FOXL2 complex formation, enhancing their binding to the *CYP19A1* promoter to directly regulate aromatase transcription in GCs. ChIP-PCR confirmed increased AR binding to three AREs in the promoter II region of *CYP19A1*, with FOXL2 overexpression further enhancing this binding. Elevated estrogen secretion was observed in GCs and cultured follicles, which was attenuated by AR knockdown or inhibition. Clinical samples showed increased estrone, estradiol, and AR, CYP19A1, and FST mRNA expression, with CYP19A1 positively correlating with AR, FOXL2, and estrogen levels.

**Conclusions:**

Hyperandrogenism in PCOS augments estrogen synthesis in GCs by enhancing AR-FOXL2 interactions, which activate *CYP19A1* gene transcription and thereby increase aromatase expression. This androgen-driven mechanism leads to elevated estrogen levels, offering new insights into the complex pathophysiology of PCOS and strengthening the rationale for targeting the AR-FOXL2-aromatase axis as a therapeutic strategy for ovulation induction in PCOS patients.

**Clinical trial number:**

Not applicable.

**Supplementary Information:**

The online version contains supplementary material available at 10.1186/s13048-025-01790-4.

## Introduction

Polycystic ovary syndrome (PCOS) is a heterogeneous disease in reproductive-aged women leading to acne, hirsutism, infertility because of biochemical hyperandrogenism, ovulatory dysfunction, and metabolic abnormalities [1]. PCOS exhibits diverse clinical features with heterogeneous nature and complexity and lacks a fully understood etiology, resulting in predominantly symptom-based and empirical treatment [2].

Hyperandrogenism emerges as the predominant endocrine aberrated hallmark of PCOS [3]. The polycystic ovary morphology can be manifested under androgen exposure in patients with adrenal hyperplasia [4], transgenders [5], and animal models [6]. Previous studies have provided strong evidence that androgens and their actions through the androgen receptor (AR) are key mediators in PCOS development and pregnancy complications [7, 8]. Considering that hyperandrogenism is the hallmark of PCOS, several androgen-treated animal models for PCOS have been generated to elucidate the etiology and development of PCOS-associated phenotypes. The most commonly used androgens in these models are testosterone, dehydroepiandrosterone, and dihydrotestosterone (DHT) [9, 10]. The timing of androgen exposure varies widely, starting as early as prenatal exposure. The clinical symptoms of PCOS often start during puberty; hence, treatment of mice before adulthood may more closely resemble human PCOS [11]. Indeed, prenatal or prepubertal androgen treatment resembles many characteristics of human PCOS, including anovulation, cystic-like follicles, elevated luteinizing hormone (LH) levels, increased adiposity, and insulin insensitivity [9]. Consequently, targeting androgen/AR axis ameliorates the chronic anovulation and menstrual cycle irregularities in PCOS animal models [12], highlighting it as a treatment regimen for hyperandrogenic PCOS [13].

Forkhead box protein L2 (*FOXL2*), a gene encoding a forkhead transcription factor, participates in the regulation of cholesterol and steroid metabolism, apoptosis, reactive oxygen species detoxification, and cell proliferation in the ovary [14]. All these recent advances indicate that FOXL2 is a central transcriptional factor required in ovarian development and maintenance [15]. Mutant *FOXL2* leads to the pathogenesis of granulosa cell tumor [16, 17]. In mice, FOXL2 is detected from 12.5 days post-coitum, and its expression continues in the granulosa cells (GCs) and stromal cells of ovarian follicles throughout the female reproductive life [18]. The importance of FOXL2 expression extends beyond this initial sex determination stage [19]; it is also required in maintaining the ovarian phenotype. The conditional deletion of the *FOXL2* gene in sexually mature female mice leads to the trans-differentiation of GCs into sertoli-like cells and the degeneration of oocytes, suggesting that FOXL2 is required to maintain GC identity in the adult ovary [20]. In the postnatal ovary, FOXL2 regulates GCs differentiation and supports follicular growth. In *FOXL2*-/- mice, GCs fail to complete the squamous-to-cuboidal transition, which represents as a gateway to GCs proliferation [18]. Without the support of cuboidal GCs, follicular growth is blocked; atresia is also observed in most of the oocytes, with no maturation into secondary follicles [19]. In mice, FOXL2 overexpression causes defects in ovary function, such as cell differentiation and steroidogenesis [21]. While most studies examined the role of FOXL2 in granulosa cell tumor pathogenesis and folliculogenesis, the roles of FOXL2 in PCOS and the relationship between AR and FOXL2 in the GCs under hyperandrogenic treatments has not yet been investigated.

In the ovary, androgens synthesized from theca cells are converted into estrogens such as estrone (E1) and 17β-estradiol (E2) in GCs by aromatase encoded by the gene cytochrome P450 family 19 subfamily A member 1 (*CYP19A1*) which is known to be regulated by FOXL2 [22]. Human aromatase is widely expressed in various tissues, including testis, ovary, placenta, bone, skin, brain, and adipose tissue, as well as benign and malignant tumors such as prostate and breast cancers [23, 24]. The human *CYP19A1* gene has nine coding exons and numerous alternative noncoding first exons used as tissue-specific promoters that regulate aromatase expression in a tissue- and cell-specific manner [25]. Tissue-specific expression of *CYP19A1* gene is achieved through the action of tissue-specific transcription factors and regulatory elements that bind to a specific promoter and enhancer regions of the gene. Promoter PII in the 5′ untranslated region of human *CYP19A1* gene is regulated by follicle-stimulating hormone (FSH) via the protein kinase A/cyclic AMP signaling pathway for proper estrogen production in GCs [26].

Elevated serum levels of E1 and free testosterone serve as reliable biomarkers with high sensitivity and specificity to distinguish women with PCOS from healthy controls [27]. Furthermore, the serum levels of E1 and E2 were both recently found to be increased in women with hyperandrogenemia PCOS compared with those in non-hyperandrogenemia PCOS women [28]. These findings are consistent with in PCOS rat models, which also displayed elevated E1 levels [29, 30]. In AR-knockout (ARKO) mice, DHT did not cause reproductive dysfunction, but testosterone treatment induced irregular cycles and ovulatory disruption [7]; thus, direct androgen actions and indirect estrogen actions converted from androgens by aromatase may be both important mediators of PCOS reproductive traits. As *CYP11A1*, *CYP17A1*, and *CYP19A1* polymorphisms are associated with hyperandrogenemia [31, 32] and dysregulation of ovarian steroidogenic enzymes in women with PCOS [33–35], this study aimed to investigate whether hyperandrogenism in PCOS may result in AR-FOXL2 axis activation to upregulate ovarian steroidogenic genes and elevate estrogen levels in GCs.

## Materials and methods

### Animals

Three-week-old C57BL/6 female mice were purchased from Biolasco Taiwan Co. Ltd. and maintained at Kaohsiung Chang Gung Memorial Hospital with 12-hour light cycle. All our study procedures were reviewed and approved by the Institutional Animal Care and Use Committee of Chang Gung Memorial Hospital.

### Generation of PCOS-like mouse models

DHT (A8380, Sigma-Aldrich, USA, 15 mg/kg/body weight) was used to induce PCOS-like traits in mice. The dosage of androgen required to induce PCOS was based on previous studies [36, 37]. Briefly, 4-week female mice were subcutaneously injected once daily with either polyethylene glycol 400 (PEG400, P3265, Sigma-Aldrich, USA) as control group or DHT dissolved in PEG400 to establish the PCOS model for 6 weeks. After complete drug administration, mice (Control group, *n* = 19; DHT group, *n* = 19, 10 mg/kg/body weight) were sacrificed to collect the ovaries and serum.

### Mouse ovary collection and follicle classification

Dissected ovaries were weighed, dehydrated, and paraffin-embedded at the pathological examination department of Kaohsiung Chang Gung Memorial Hospital. They were serially sectioned at 5 μm. The classification of preantral follicles (oocyte with 2–5 layers of cuboidal GCs), small antral (oocyte surrounded with > 5 layers of GCs, and 1 or 2 small areas of follicular fluid), large antral follicles (contained 1 large antral cavity) was based on a previous study [38]. For all large antral follicles, the thickness of the granulosa layer and theca layer was measured using Image J software (RRID: SCR_003070).

### Single-cell RNA-sequencing analysis and data processing

Transcriptomic data of ovaries collected from control mice and PCOS mice performed through Illumina platform was obtained from GSE268919. Mouse genome (GRCm38/mm10) was sourced from UCSC Genome Browser (https://genome.ucsc.edu). Cell Ranger (RRID: SCR_023221) and Loupe Browser (RRID: SCR_018555) were utilized in processing and aligning Single-cell RNA-sequencing (scRNA-seq) data. Cell type definition were determined by previous study [39] and annotated manually. UMAP was used for visualization of cell type distribution and relationships.

### Mouse follicle isolation and in vitro culture

Ovaries were collected from 3-week-old C57BL/6 female mice. Large preantral follicles (LPAFs, 110–170 μm in diameter) with intact GCs layers, round oocyte, and basement membrane were mechanically isolated from dissected ovaries by using acupuncture needles, as previously described [40]. We used the α-minimum essential medium (12571063, Gibco, USA) with 10% charcoal/dextran-treated fetal bovine serum (CD-FBS, 12676029, Gibco, USA), recombinant human FSH (10 ng/ml, Gonal-F, Merck, Germany), insulin (10 mg/L), transferrin (5.5 mg/L), sodium selenite (6.7 µg/L, Corning, USA), penicillin (100 units/ml), and streptomycin (100 mg/L; 15240096, Gibco, USA) to cultivate follicles in 96-well plates (1 follicle/well). Follicles were cultured under humid conditions at 37 °C with 5% CO_2_, and half of the medium would change every 2 days. LPAFs (8 follicles/mouse/group) from mice were randomly divided into two groups: VE (medium with ethanol for 48 h) and DHT (medium with 1 µM DHT for 48 h).

### Isolation of mouse primary GCs

The 3-week-old mouse ovaries were washed with phosphate buffer saline (PBS). Then, minced tissues were digested with collagenase (1 mg/ml; S1746501, Nordmark, Germany) in 0.25% trypsin (15050065, Gibco, USA) and centrifuged at 1000 rpm and 37 °C for 60 min. Furthermore, mouse primary GCs (MPGCs) were separated by filtration using a 40 μm pore size nylon mesh. Filtered MPGCs were allowed to attach on a dish overnight, and then blood cells and tissue debris were washed away with PBS. MPGCs were verified by the IF staining of the FSH receptor (FSHR) antibody (Fig. S3).

### Cell culture, overexpression, and knockdown

Human primary GCs, MPGCs, and KGN (RRID: CVCL_0375, RCB1154, RIKEN BRC Cell bank) were all cultured in DMEM/F12 medium with 10% FBS (10437028, Gibco, USA) in a humidified atmosphere with 5% CO_2_ at 37 °C. KGN was transiently overexpressed with FOXL2 (pCS2-flag-FOXL2, 153135, Addgene, Cambridge, MA, USA) via Lipofectamine™ 2000 Transfection Reagent (11668019, Thermofisher, USA) according to the manufacturer’s instructions. AR in KGN was knocked down by two lentivirus packaging AR-shRNA cloning units (sh-AR-1, TRCN0000350462; sh-AR-2, TRCN0000314730) from Academia Sinica Institutional Animal Care and Utilization Committee (Taipei, Taiwan). The number of viral particles was optimized to infect KGN at a multiplicity of infection of 2–3 for 3 days. After 3 days of 1 µg/ml puromycin selection, KGN was cultured in fresh medium and prepared for the subsequent experiments.

### RNA extraction and quantitative RT-PCR

Total RNA was extracted from cells and grinded tissue by using the TRIZOL Reagent. Then, the RNA was reverse-transcribed in 2–5 µg of the total, using the reverse transcriptase kit (PRA5000, Promega, USA). Next, we incubated the RNA samples at 70 °C for 5 min, immediately chilled in ice water for at least 5 min, and combined with Reverse Transcription Mix. The samples were subsequently incubated at 25 °C for 5 min and at 42 °C for 60 min, and reverse transcriptase was inactivated by heating at 70 °C for 15 min and cooling at 4 °C. The reaction products were stored at 4 °C in the reaction tubes or wells for immediate analysis (up to 24 h) or at − 20 °C for long-term storage. We performed RT-PCR in Fast SYBR green master mix kit (4385612, Applied Biosystems, USA). Sequences were analyzed using an ABI 7500 Fast sequence detection system. Supplementary Table 1 lists all the sequences of all primers used in this study. We quantified transcripts of the gene β-actin as the endogenous RNA control and normalized each sample on the basis of its β-actin content. Relative transcription levels were calculated using 2^−ΔΔCT^ methods.

### ELISAs for E1 and E2 quantification

Seeded 10^4^/cm^2^ KGN and 5 × 10^4^/cm^2^ MPGCs were cultivated in phenol red-free DMEM/F12 medium (21041025, Gibco, USA) with 10% CD-FBS and 1 nM testosterone (86500, Sigma-Aldrich, USA). Furthermore, 10 mouse LPAFs seeded in 96-well plates were cultivated in phenol red-free α-minimum essential medium (41061029, Gibco, USA) with 10% CD-FBS and the necessary supplements as mentioned above. To imitate the hyperandrogenic environment, we treated the cells and follicles with 1 µM androgens (DHT or testosterone) for 48 h. The collected culture media were utilized to measure estrogen levels using the estrone ELISA kit (EU3107, Finetest, China) and 17β-estradiol ELISA kit (ADI-901-008, Enzo, USA).

### Immunohistochemical, and immunofluorescence staining

Formalin-fixed and paraffin-embedded mouse ovarian sections or seeded GCs underwent immunohistochemica (IHC), and immunofluorescence (IF) staining. The tissue sections were deparaffinized and rehydrated before staining. Regarding the IHC staining, the sections were blocked with 3% hydrogen peroxide blocking reagent for 15 min to deprive the endogenous peroxidase activity, microwaved in 10 mM citrate buffer at pH 6.0 to unmask the epitopes, and then incubated with the primary antibodies overnight in a humidified chamber at 4 °C. After incubation with the secondary IgG antibody, the sections were stained with 3,3′-diaminobenzidine (DAB, Dako, Glostrup, Denmark) and counterstained with hematoxylin. 5 to 7 ovaries were used in each groups. The intensity of DAB positive staining (H score) in IHC was quantified by IHC profiler (RRID: SCR_023577). H score = (1 × % Low Positive) + (2 × % Positive) + (3 × % High Positive), giving a total score between 0 and 300. As for the IF staining, the sections were incubated overnight with the primary antibody in a humidified chamber at 4 °C after being treated with 10% horse serum to block any nonspecific antibody binding. Moreover, primary antibody binding was visualized using Alexa Fluor^®^ 488 Goat Anti-Rabbit IgG (H + L) (A11034, Invitrogen, USA) and Alexa Fluor^®^ 568 Goat Anti-Mouse IgG (H + L) (A11031, Invitrogen, USA). Finally, the sections were mounted with a mounting medium containing 4’,6-diamidino-2-phenylindole (DAPI) (Vector Laboratories). The images of immunostained FSHR were captured using the Leica DM3000 fluorescence microscope (Leica, Wetzlar, Germany), and the images of double-immunostained AR and FOXL2 were captured using the Olympus FV 1000 confocal microscope system and analyzed with the Olympus Fluoview version 2.1 C software (Olympus Corporation, Tokyo, Japan). Supplementary Table 2 lists all antibodies used in IHC and IF staining.

### Western blotting

Cells was lysed and sonicated in Radio-Immunoprecipitation Assay buffer, and protein concentration was determined by the Bradford assay. Furthermore, 30 µg of protein was loaded into sodium dodecyl sulfate–polyacrylamide gel electrophoresis and then transferred to nitrocellulose membranes (Amersham Biosciences, USA). The membranes were blocked by 5% nonfat milk/Tris-buffered saline with Tween^®^ 20 Detergent for 1 h and probed with the primary antibody at 4 °C overnight. After membrane washing, the secondary antibody was used for incubation for 1 h, and immunodetection was conducted using ECL (PerkinElmer™, USA). Supplementary Table 2 presents a list of all antibodies used in Western blotting.

### Molecular docking

The AR-FOXL2 interaction was predicted using HDOCK, a web server [41]. Additionally, polar contacts of amino acid residues of the AR and FOXL2 were found, followed by mapping using PyMOL 3.0 (RRID: SCR_000305).

### Immunoprecipitation

MPGCs lysates were prepared as described above, and protein lysates (500 µg/500 µl) containing 50 µl of magnetic beads (LSKMAGA02, Millipore) were incubated with anti-AR antibody (06-680, Millipore), anti-FOXL2 antibody (ab246511, Abcam, UK), or rabbit IgG (sc-2027, Santa cruz, USA) overnight at 4 °C for immunoprecipitation (IP). Under magnetic bead purification system, beads were washed with PBS, and denatured proteins were collected after boiling. These proteins were then used for Western blotting.

### Prediction of androgen receptor response element (ARE) in the promoter II region of CYP19A1 gene

We used the Genome Browser data hub UCSC (http://genome.ucsc.edu/) to predict the possible AREs in the *CYP19A1* promoter II region, and the JASPAR transcription factor database (http://jaspar.genereg.net/) to analyze the presence of AR-binding sites in this region.

### Chromatin immunoprecipitation

We used an EZ-Magna ChIP assay kit (MAGNA0017, Millipore, USA) to conduct Chromatin immunoprecipitation (ChIP) according to the manufacturer’s instructions. KGN was cross-linked in 1% formaldehyde and then sonicated. After centrifugation, cell lysates containing immune complexes were incubated with anti-AR (5153 S, Cell Signaling Technology, Boston, MA, USA), anti-RNA polymerase II (05-623B, Millipore, USA), or normal IgG at 4 °C overnight with rotation. We kept 1% of diluted lysates for the DNA input group. After the immune complexes were washed thrice with washing buffer, the DNA fragments were eluted and analyzed using PCR.

### Luciferase reporter gene assays

We seeded 5 × 10^3^/cm^2^ KGN cells 18 h before transient transfection with constructs of pGL4-*CYP19A1*-promoter II-589 bp-luciferase. Dual-Luciferase Reporter Assay System (E1910, Promega, USA) was applied to determine the ratio of firefly luciferase activity to Renilla luciferase activity in the samples to represent the biological activity of luciferase reporter constructs.

### Clinical sample selection

The study was approved by the Ethics Committee of Chang Gung Memorial Hospital and conducted at An-Ten Obstetrics and Gynecology Clinic. Samples were obtained from patients who provided informed consent between September 2023 and December 2023 (IRB approval number: 202301022B0). PCOS diagnosis was based on the 2003 Rotterdam diagnostic criteria, which require two of the following three manifestations: (1) oligo- and/or anovulation, (2) hyperandrogenism, and (3) polycystic ovaries morphology. We selected 13 patients for the PCOS group determined by the Rotterdam diagnostic criteria with hyperandrogenemia. These patients then received the standard in vitro fertilization (IVF) flexible-start antagonist stimulation protocol. For the control group, we selected another 13 women who underwent IVF treatment with regular menstrual cycles, normal sex hormone levels, and normal ovarian morphology. Patients who had adrenal hyperplasia, Cushing’s syndrome, and thyroid diseases were excluded. The blood and follicular fluid samples were collected from the participants during the oocyte retrieval surgery (approximately 35–36 h after administering the human chorionic gonadotropin/gonadotropin-releasing hormone agonist). The body mass index (BMI) values, hormonal, biochemical parameters of the control and PCOS groups were documented. Table 1 summarizes patients’ clinical information. The follicular fluid of the largest, first punctured follicle was collected. All the selected follicles were surrounded by the cumulus-oocyte complexes. The ovarian follicles were aspirated independently. Human primary GCs were isolated from the participants’ follicular fluid samples by using histopaque Ficoll 1077 (10771, Merck, Germany). First punctured follicular fluid was used to evaluate E1 and E2 levels via estrone enzyme-linked immunosorbent assay (ELISA) (EU3107, Finetest, China) and 17β-estradiol ELISA (ADI-901-008, Enzo, USA), respectively. Human primary GCs from patients with PCOS were further verified by IF staining with FSHR antibody (Fig. S3).

### Statistical analysis

Experiments were repeated independently at least thrice. Data were presented as mean ± standard error of the mean (SEM) via GraphPad Prism 6 (RRID: SCR_002798). For parametric tests, the statistical significance between two groups was determined by two-tailed Student’s t test and three or more groups was evaluated by one-way analysis of variance (ANOVA) with Bonferroni’s test. A *p*-value less than 0.05 was considered statistically significant.

## Results

### Comparison of AR, and FOXL2 and their downstream gene expression profiling in PCOS models

**Fig. 1:**
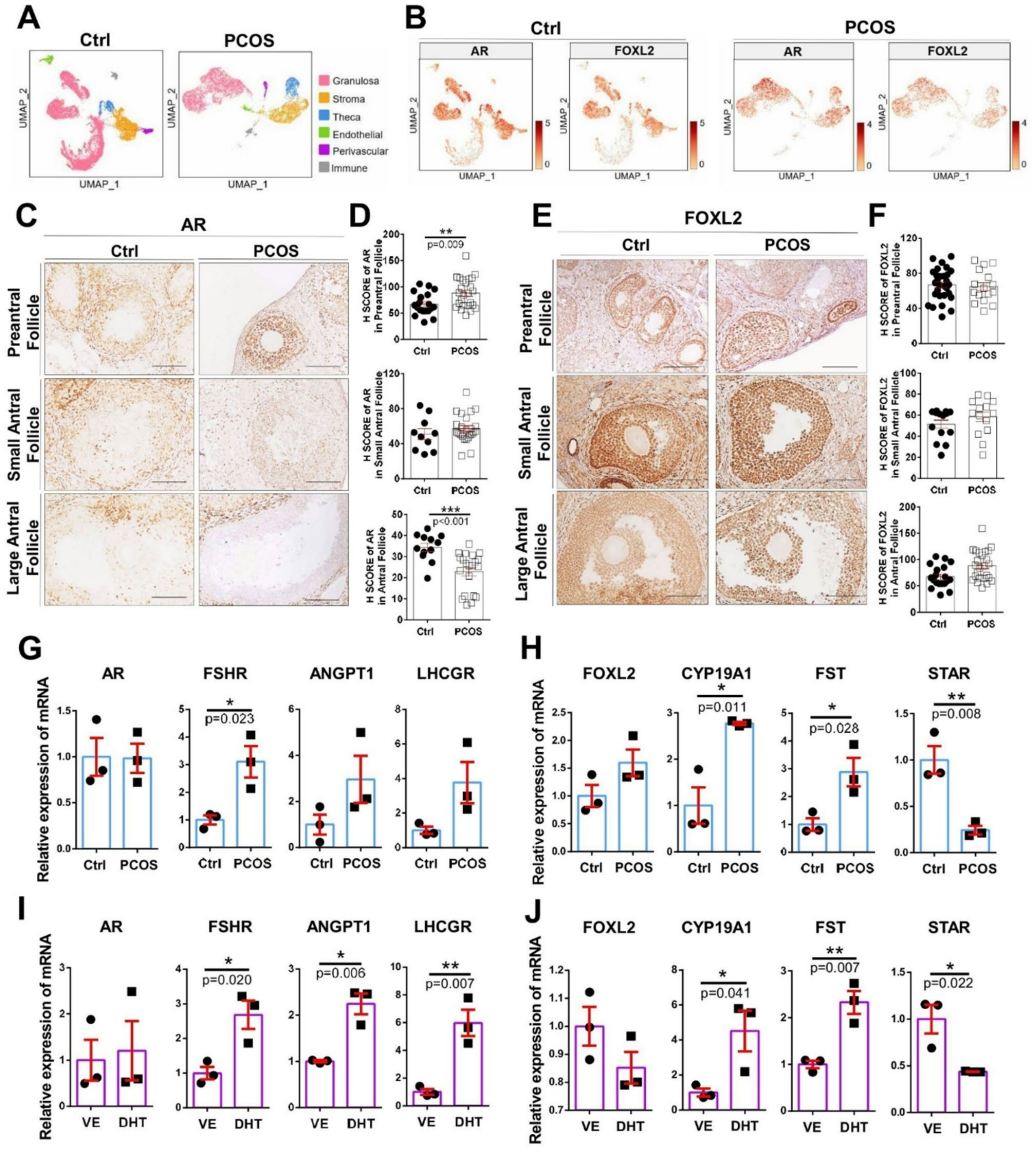
AR and FOXL2 downstream genes were activated in both PCOS mouse ovaries and DHT-treated MPGCs. (**A**) Distinct cell type patterns of control and PCOS mouse ovarian populations were classified into granulosa cells, thecal cells, stroma cells, perivascular cells, endothelial cells, and immune cells with different colors by scRNA-seq. (**B**) Feature plots showed similar expression profiles of AR and FOXL2 within the control and PCOS populations by UMAP visualization. (**C**) AR and (**E**) FOXL2 protein expression patterns in the different follicles of ovary samples isolated from the control and PCOS mice by immunohistochemistry staining (*n* = 10–29). (**D**) AR and (**F**) FOXL2 protein expression intensity in preantral follicles, small and large antral follicles was quantified by H score. Bars represent 100 μm. (**G**) Relative mRNA expression levels of AR and its downstream genes (FSHR, ANGPT1, and LHCGR), (**H**) FOXL2 and its downstream genes (CYP19A1, FST, and STAR) in ovarian samples from control and PCOS mice (*n* = 3). (**I**) Expression profiles of AR and its downstream genes, (**J**) FOXL2 and its downstream genes in MPGCs cultured for 72 h in the absence or presence of DHT (*n* = 3). Data were analyzed by unpaired *t*-test and were expressed as the mean ± SEM *, *p* < 0.05; **, *p* < 0.01; ***, *p* < 0.001. Ctrl, control group (ethanol-treated); VE, vehicle group (ethanol-treated)

To investigate to investigate the expression patterns and downstream signaling pathways of AR and FOXL2 which may play crucial roles in the hyperandrogenic characteristic of PCOS, scRNA-seq dataset was used to establish cell-specific gene expression patterns across ovarian cell populations. The cell population from control and PCOS-like mouse models were further clustered into six subgroups (Fig. 1A, S1). Granulosa cells, thecal cells, stroma cells, perivascular cells, endothelial cells, and immune cells was classified by subpopulation-specific markers followed by the previous study [39]. Notably, feature plots demonstrated that both AR and FOXL2 exhibit similar expression patterns across ovarian cells including granulosa cells, thecal cells, stroma cells populations (Fig. 1B), indicating potential functional interplay between AR and FOXL2 signaling pathways in regulating the follicular development of ovarian physiology and PCOS pathology.

**Fig. 2:**
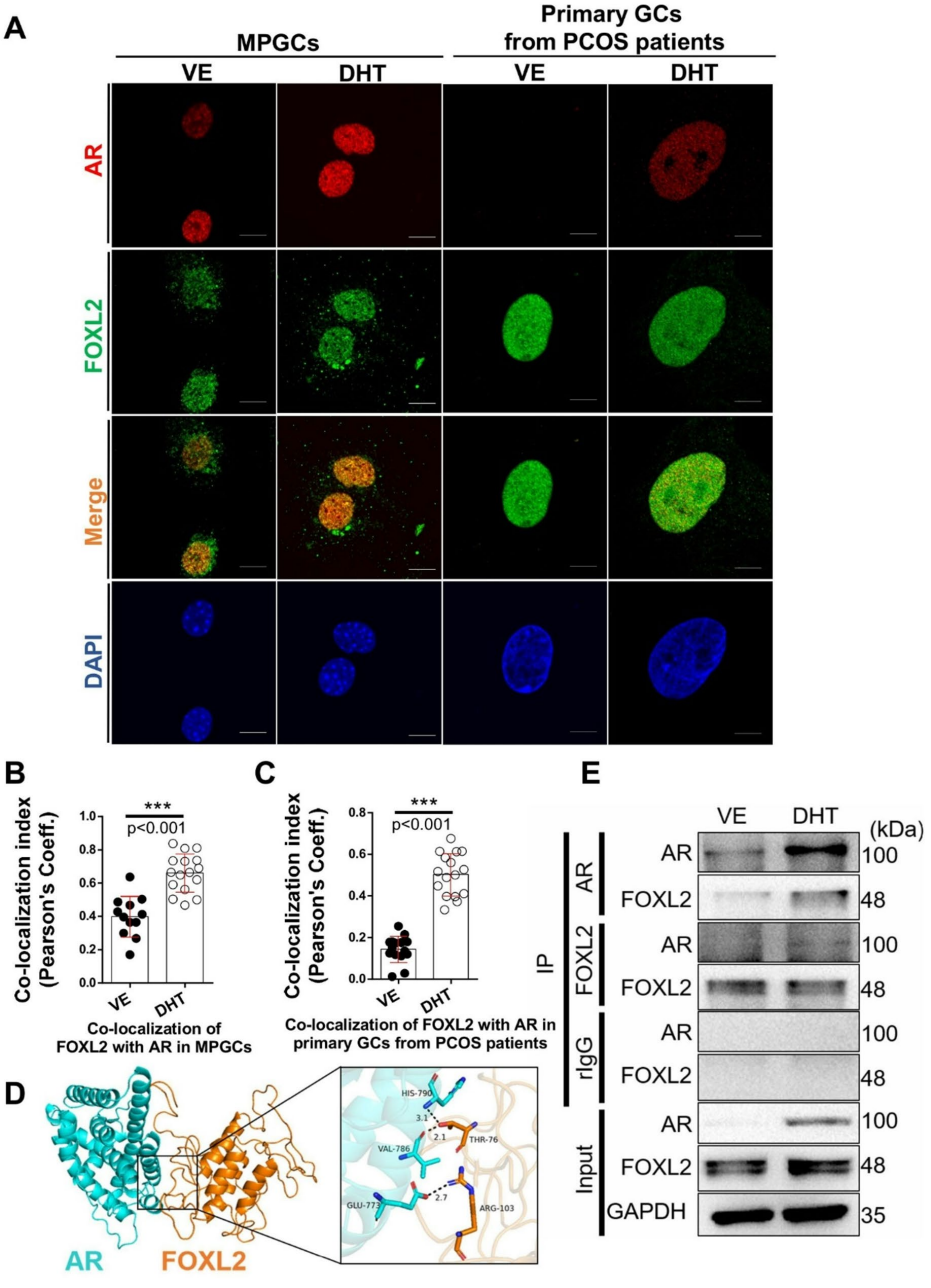
Co-localization and interaction of FOXL2 and AR in MPGCs and primary GCs from patients with PCOS. (**A**) FOXL2 and AR were labeled with Alexa488 (green) and Alexa568 (red), respectively. DAPI was counterstained for nuclei. Bars represent 10 μm. Pearson’s coefficient measured the co-localization of AR and FOXL2 in ethanol (VE)- and DHT-treated (**B**) MPGCs and (**C**) primary GCs from patients with PCOS (*n* = 12–16). Data were expressed as the mean ± SEM; ***, *p* < 0.001. Unpaired *t*-test was conducted under 3 independent experiments. (**D**) Representative images of the predicted docking model of AR-FOXL2 interaction with the possibility of forming 3 hydrogen bonds (AR-E773, V786, and H790; FOXL2-T76, and R103), and the distance of hydrogen bonds (angstroms). (**E**) Western blotting of the input and immunoprecipitated samples with anti-AR (IP: AR), anti-FOXL2 (IP: FOXL2), and anti-rabbit IgG (IP: rIgG) antibodies showed the co-IP of FOXL2 with AR and co-IP of AR with FOXL2

**Fig. 3:**
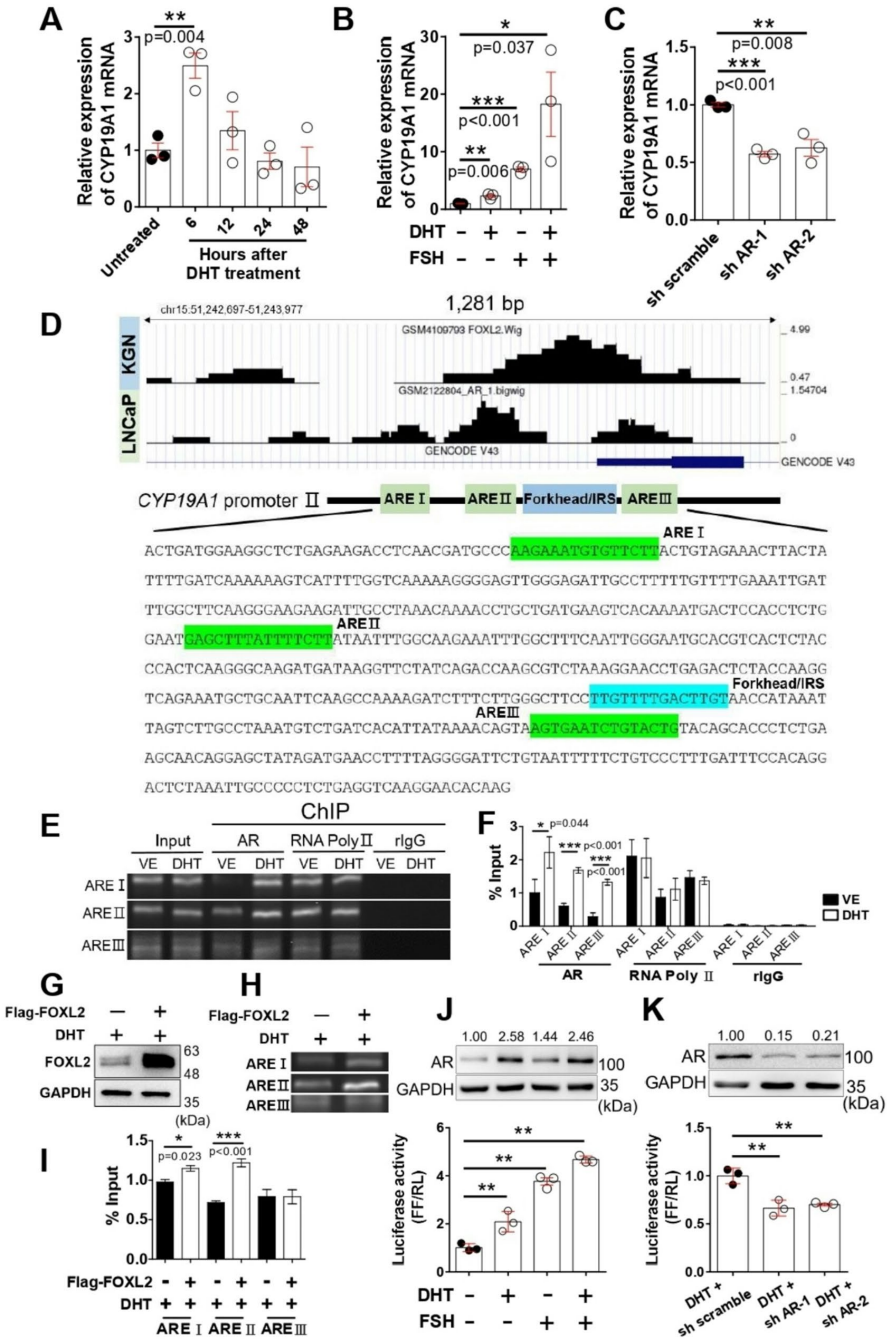
Androgen induced CYP19A1 promoter activation. Relative expression of CYP19A1 mRNA in KGN (**A**) treated with 1 µM DHT for 6, 12, 24, and 48 h (*n* = 3) and (**B**) treated with 1 µM DHT for 6 h and/or 100 ng/ml follicle-stimulating hormone (FSH) for 48 h only (*n* = 3). (**C**) Relative expression of CYP19A1 in KGN with AR knockdown (*n* = 3). (**D**) Putative androgen receptor response elements (AREs) were predicted by UCSC and JASPAR and annotated the human aromatase promoter II (589 bp) sequence cloned on pGL4-luciferase vector. Chromatin immunoprecipitation (ChIP)-PCR was performed in KGN by using AR antibody to quantify the binding of AR on *CYP19A1* gene locus. (**E**) The representative PCR analysis for each putative ARE and (**F**) the quantitative results (*n* = 4). ChIP-PCR was also conducted in KGN to test whether AR binding on *CYP19A1* gene locus would be increased by FOXL2 overexpression. (**G**) The result of western blotting showed the overexpression of FOXL2 in KGN; (**H**) The representative PCR analysis for 3 AREs and (**I**) the quantitative results (*n* = 3). (**J**) The upper panel showed AR protein expression in KGN treated with 1 µM DHT and/or 100 ng/ml FSH under a serum-free condition for 24 h; the lower panel showed luciferase assay with the same treatment condition for 24 h (*n* = 3). (**K**) The upper panel showed AR expression in AR knockdown KGN treated with 1 µM DHT for 24 h; the lower panel showed luciferase assay with the same treatment condition for 24 h (*n* = 3). Data of mRNA expression and ChIP were analyzed by unpaired *t*-test and Data of luciferase assay were analyzed by one-way ANOVA with the mean ± SEM; *, *p* < 0.05; **, *p* < 0.01; ***, *p* < 0.001

**Fig. 4:**
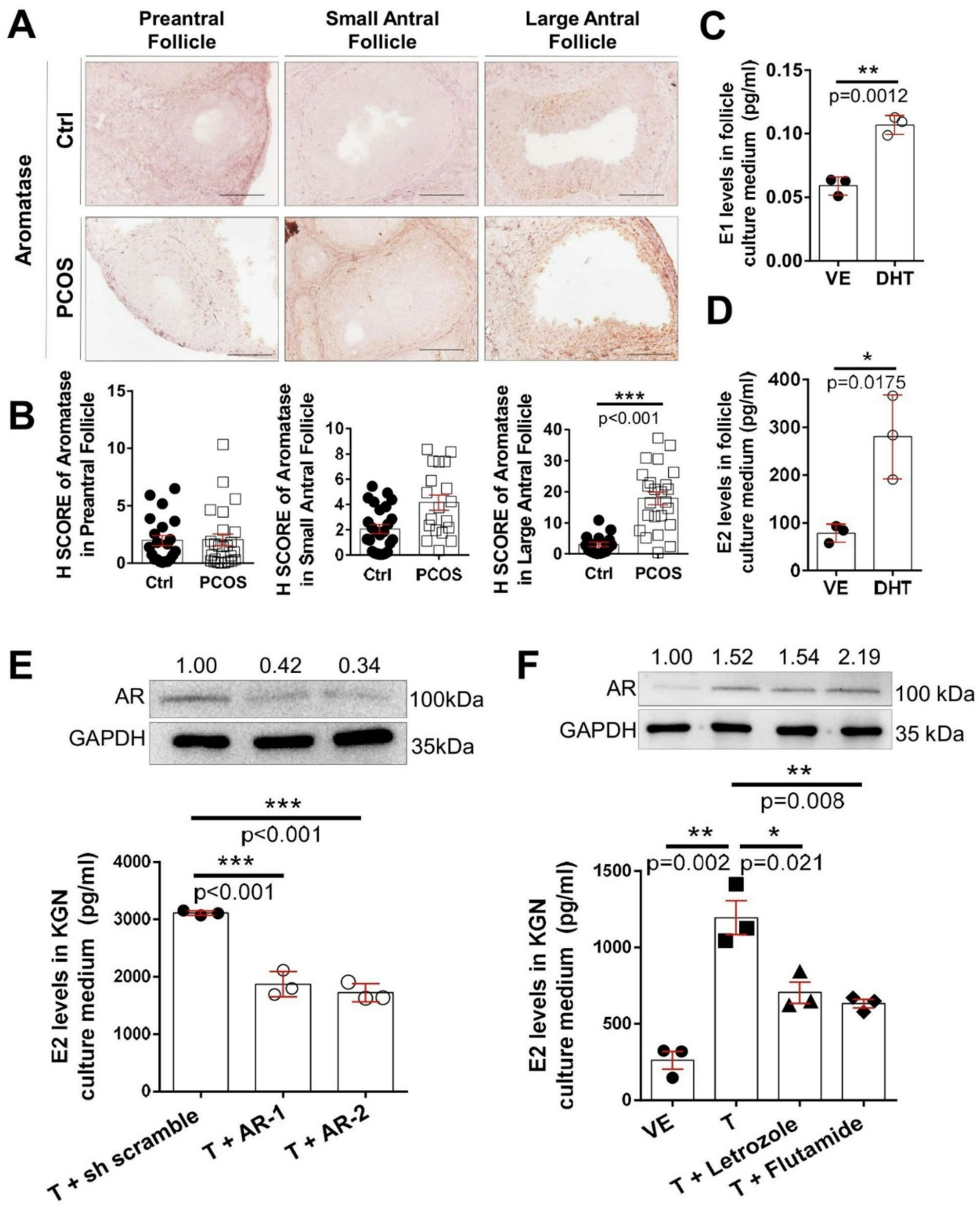
Androgen excess increased estrogen levels in follicles and GCs. Expression of aromatase in the follicle of hyperandrogenic mice. (**A**) Representative follicles showing aromatase staining results in the ovary isolated from the PCOS mouse model. (**B**) H score was used to quantify aromatase intensity in preantral follicles and both small and large antral follicles (*n* = 18–25). Bars represent 100 μm. The levels of (**C**) estrone and (**D**) 17β-estradiol (E2) in follicle culture medium (*n* = 3). (**E**) The upper panel showed AR protein expression in AR knockdown KGN, the lower panel showed the levels of E2 in AR knockdown KGN culture medium (*n* = 3). (**F**) The upper panel showed AR protein expression in drug-treated KGN, the lower panel showed the levels of E2 in KGN treated with 100 nM letrozole or 100 nM flutamide culture medium for 48 h (*n* = 3). Data were analyzed by unpaired *t*-test with the mean ± SEM; *, *p* < 0.05; **, *p* < 0.01; ***, *p* < 0.001

**Fig. 5:**
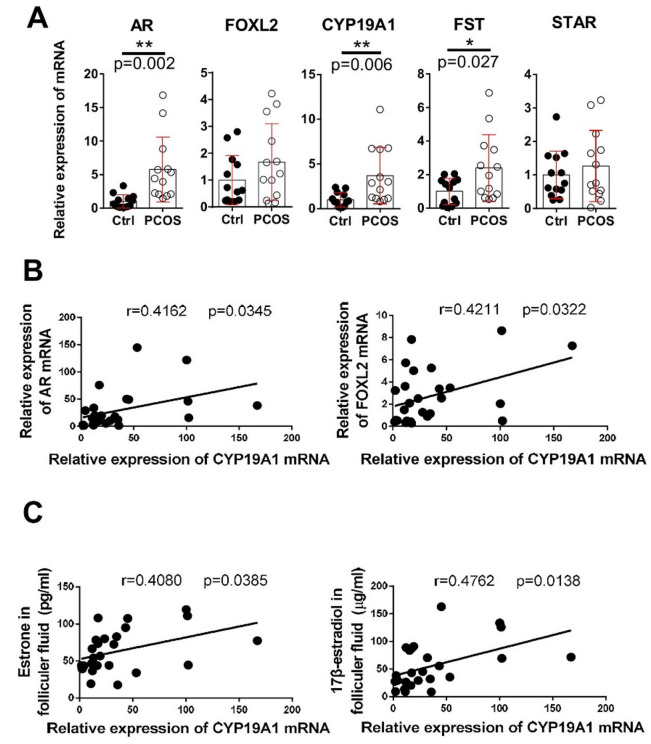
Relative expression of AR, FOXL2, and FOXL2 downstream genes and correlation between CYP19A1 and AR, and FOXL2 and estrogens in human GCs from control patients (*n* = 13) and patients with PCOS (*n* = 13). (**A**) Quantification of the mRNA levels of AR, FOXL2, CYP19A1, FST and STAR. Data were analyzed by unpaired *t*-test with the mean ± SEM; *, *p* < 0.05; **, *p* < 0.01. Association of the relative mRNA expression of CYP19A1 in human GCs with that of (**B**) AR, FOXL2, and (**C**) the levels of estrone and 17β-estradiol in human follicular fluids

**Fig. 6:**
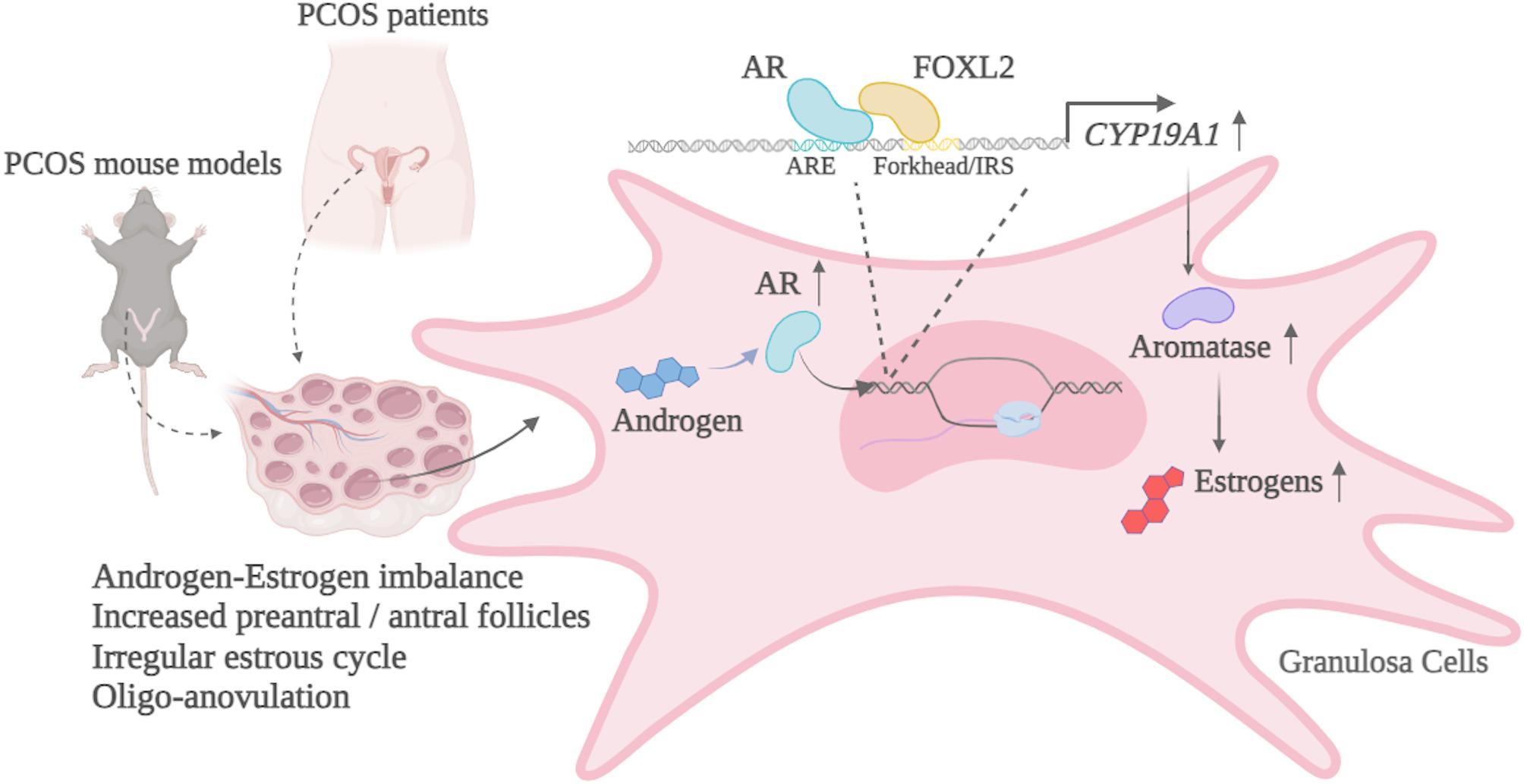
A schematic graph highlights the effects of hyperandrogenism and the roles of AR and its interaction with FOXL2 through binding to androgen receptor response element (ARE) and forkhead/insulin response sequences in the promoter regions of *CYP19A1* gene to induce *CYP19A1* gene transcription, thereby increasing aromatase protein expression and estrogen production. The resulting androgen–estrogen imbalance appears to be a critical factor in PCOS pathogenesis, likely contributing to PCOS’ hallmark features, including increased preantral and antral follicles, irregular estrous cycles, and oligo-anovulation

**Table 1:** Demographic characteristics and relative gene expression of recruited women with and without PCOS

	Control group	PCOS group	*p* value
Age (year)	33 ± 5	31 ± 3	0.140
BMI (kg/m^2^)	21.5 ± 2.0	25.9 ± 5.7	0.015 *
E1 in follicular fluid (pg/ml)	47.87 ± 19.55	78.10 ± 29.08	0.005 **
E2 in follicular fluid (µg/ml)	24.28 ± 11.46	78.66 ± 35.55	< 0.001 ***
AMH in serum (ng/ml)	2.49 ± 1.12	6.60 ± 0.85	< 0.001 ***
T in serum (ng/ml)	0.50 ± 0.31	0.93 ± 0.50	0.017 *
Number of oocytes retrieved	10 ± 5	29 ± 10	< 0.001 ***

To further characterize androgen effects on the underlying AR and FOXL2-mediated downstream-targeted genes in mouse ovaries and MPGCs, we first performed immunohistochemistry staining to evaluate AR and FOXL2 protein expression in different follicle types (preantral, small antral, and large antral) from isolated mouse ovaries. The preantral follicles in the ovaries of PCOS mouse models exhibited higher AR expression than those in control mouse ovaries (Fig. 1C and D). In contrast, the large antral follicles in ovaries from PCOS mouse models showed lower AR expression than those from control mice (Fig. 1D). FOXL2 expression was not significantly different between preantral and antral follicles, regardless of the presence of androgen (Fig. 1E, F). However, clustering analysis of differentially expressed genes showed that FOXL2 downstream genes associated with folliculogenesis, and steroidogenesis were distinctly expressed in the ovaries of PCOS mice compared to those of control mice (Fig. S2). By using RT-PCR, we further analyzed the mRNA expression of AR and its downstream genes, including *FSHR*, angiopoietin 1 (*ANGPT1*), and LH/choriogonadotropin receptor (*LHCGR*), which play critical roles in ovarian function [42]. The mRNA expression of these genes was significantly upregulated in PCOS ovaries (Fig. 1G), follicles (Fig. S4A), and DHT-treated MPGCs (Fig. 1I). Subsequently, the mRNA expression levels of *FOXL2*, FOXL2-activated genes (*CYP19A1* and *FST*) and repressed gene (*STAR*) were analyzed. Interestingly, the mRNA expression of *CYP19A1* and *FST* was upregulated, while that of STAR was downregulated in PCOS ovaries (Fig. 1H) and MPGCs with DHT treatment (Fig. 1J). Similarly, *CYP19A1* gene was also upregulated in antral follicles in the presence of DHT treatment (Fig. S4B).

### AR was co-localized and interacted with FOXL2 in GCs

While FOXL2 downstream genes were regulated by DHT, the relationship between AR and FOXL2 in GCs remains unknown. To explore the interaction between FOXL2 and AR, we first used double IF staining with specific antibodies to detect AR and FOXL2 localization in MPGCs and human primary GCs. Both AR and FOXL2 were expressed in the nucleus of MPGCs and human primary GCs. By using Pearson’s coefficient analysis, DHT treatment significantly increases co-localization of AR and FOXL2 compared to vehicle treatment. (Fig. 2A-C). In Fig. 2D, a molecular docking model was established to predict the physical interaction between AR and FOXL2 proteins and highlight potential interaction sites with hydrogen bonds between specific amino acid residues: AR (E773, V786, and H790) and FOXL2 (T76 and R103). The co-immunoprecipitation analysis further used to confirm the interaction between AR and FOXL2 in MPGCs. When AR is immunoprecipitated (IP: AR), FOXL2 is pulled down, confirming their physical association. Similarly, when FOXL2 is immunoprecipitated (IP: FOXL2), AR is pulled down in the DHT-treated condition compared to vehicle treatment. (Fig. 2E). This DHT-dependent manner suggests that androgen signaling enhances the interaction between these two proteins, providing a potential molecular mechanism for how hyperandrogenism affects granulosa cell function in PCOS.

### FOXL2 coordinates with AR to activate CYP19A1 gene transcription and increases Estrogen levels in GCs

Taking into account that CYP19A1 mRNA increased under hyperandrogenism treatment, we hypothesized that AR may bind to the promoter of *CYP19A1* gene to regulate aromatase expression. Thus, we constructed a reporter gene containing *CYP19A1* promoter PII region sequences to measure the AR transcriptional activity. Indeed, CYP19A1 mRNA expression was significantly increased in KGN with DHT treatment for 6 h (Fig. 3A). Considering that FSH regulates CYP19A1 mRNA by activating its promoter II transcriptional activity [43], we examined whether DHT-induced CYP19A1 mRNA expression can be enhanced in the presence of FSH. As shown in Fig. 3B, the DHT-induced mRNA expression of *CYP19A1* gene was further augmented by FSH treatment. Conversely, the AR knockdown in KGN by AR-shRNA reduced CYP19A1 mRNA expression (Fig. 3C). To further investigate the underlying mechanism of AR-FOXL2 interaction on regulating the promoter of *CYP19A1* gene under hyperandrogenism, we further analyzed the ChIP-seq data of FOXL2 and AR-binding peaks on human *CYP19A1* gene by using the Genome Browser data hub UCSC and JASPAR. We identified three putative AREs closed to the FOXL2-binding site in the promoter II region of the human *CYP19A1* gene (Fig. 3D). Indeed, ChIP-PCR results demonstrated that DHT increased AR binding to three AREs in the promoter II region of the human *CYP19A1* gene (Fig. 3E, F). To further investigate whether FOXL2 can enhance AR binding to the ARE regions of the promoter of *CYP19A1* gene, we overexpressed FOXL2 in KGN (Fig. 3G). Subsequently, ChIP-PCR results demonstrated that FOXL2 increased the binding ability of AR with AREI and ARE II sites in the *CYP19A1* promoter PII region (Fig. 3H, I). The transcriptional activity of *CYP19A1* gene promoter II was also activated in the presence of DHT and FSH (Fig. 3J) but was suppressed by AR-shRNA (Fig. 3K) in luciferase assay. Therefore, the AR-FOXL2 interactive complex may bind to the promoter II region of the human *CYP19A1* gene to activate its transcription, and it may increase the mRNA expression of CYP19A1.

Further IHC staining results indicated that DHT, exclusively enhanced aromatase expression in large antral follicles but not in preantral and small antral follicles (Fig. 4A, B). In parallel, we collected an in vitro culture medium from androgen-treated mouse follicles and examined MPGCs and KGN to determine whether excess androgens can enhance the estrogen levels. The concentrations of E1 and E2 were elevated in the culture medium of mouse follicles with or without testosterone and DHT (Fig. 4C, D). Similarly, the E2 level was increased in testosterone- and DHT-treated MPGCs (Fig. S5A) and KGN (Fig. S5B). Conversely, testosterone-induced E2 level was suppressed by the knockdown of AR, letrozole (an aromatase inhibitor), and flutamide (an androgen antagonist) (Fig. 4E and F).

### Estrogen production and CYP19A1 gene expression are increased in patients with PCOS

To explore the differences in the hormone imbalance and characterize the roles of AR and FOXL2 in patients with PCOS, we analyzed the PCOS-related hormone levels in follicular fluids and the expression of AR, FOXL2, and FOXL2 downstream genes, including *CYP19A1*, *FST* (follistatin), and *STAR* (steroidogenic acute regulatory protein) in human primary GCs. Compared with the control group, the PCOS group exhibited not only increases in anti-Müllerian hormone (AMH) and testosterone levels in serum but also increases in E1 and E2 levels in follicular fluids (Table 1). In addition, the mRNA expression of AR, CYP19A1, and FST were upregulated in GCs from patients with PCOS (Fig. 5A). Further regression analysis showed that increased CYP19A1 mRNA levels in the GCs did not only positively correlate with increased FOXL2 and AR mRNA levels (Fig. 5B) but also positively correlated with elevated E1 and E2 levels in human follicular fluids (Fig. 5C).

## Discussion

PCOS is a complex endocrine disorder affecting reproductive-age women. In this study, we aimed to investigate the effects and molecular mechanisms of hyperandrogenism on GCs dysfunction underlying PCOS. Using both PCOS patient samples in vitro cell and in vivo mouse models, we revealed that excess androgens differentially impact the regulation of steroidogenesis and folliculogenesis in the ovary, with one key regulatory pathway involving AR directly interacting with FOXL2. This interaction promotes *CYP19A1* gene transcription, leading to increased aromatase expression and estrogen synthesis in GCs. (Fig. 6). These findings elucidate a novel mechanism whereby hyperandrogenism augments the GCs-specific regulation of estrogen synthesis via AR-FOXL2–mediated activation of the aromatase gene in PCOS.

The etiology of PCOS remains unknown, but androgen excess is widely accepted as the basis of this syndrome. Activation of androgen/AR signaling plays a crucial role in PCOS pathogenesis [7]. The administration of the AR blocker, flutamide, not only prevents reproductive dysfunction in PCOS mice [8], but attenuates accelerated lipid accumulation in PCOS patients [44], likely by reducing androgenic signaling. In addition to hyperandrogenism, hormonal imbalances in PCOS including elevated LH, AMH, prolactin, and inhibin levels [45]. E2, is considered the most potent estrogen in humans and exerts effects via estrogen receptor 1(ESR1), estrogen receptor 2 (ESR2) and the G-protein-coupled estrogen receptor [46]. E2 plays a crucial role in female reproductive function and may contribute to PCOS development, as suggested by polycystic ovarian morphology found in estradiol valerate–treated rats [47, 48]; however, its clinical utility as a diagnostic marker for PCOS is limited because of variable ovulatory patterns. Instead, increased serum E1 levels converted from increased androstenedione levels by aromatase was consistently observed in PCOS [27, 45]. Moreover, E1 level is significantly correlated with serum LH and androgen levels in women with PCOS [45, 49]. Consistent with previous findings [50, 51], our study revealed that both E1 and E2 levels were significantly increased and positively correlated with CYP19A1 mRNA expression in GCs (Fig. 5C). The CYP19A1 mRNA levels were also correlated with FOXL2 and AR mRNA levels in the human GCs (Fig. 5B), suggesting a potential regulatory relationship among these genes to coordinate the regulation of steroidogenesis including estrogen production in GCs.

The activation of androgen-AR pathway significantly impacts metabolism in GCs from women with PCOS, disrupting follicle growth and oocyte maturation [52]. AR expression has been significantly associated with the number of follicles in the ovaries of patients with PCOS [53]. Within follicles, AR is predominantly expressed in the granulosa and theca cells, where it regulates steroidogenesis and follicular development. Our results showed that the AR protein levels were apparently increased in early-stage follicles but significantly declined in large antral follicles (Fig. 1C and D), consistent with previous findings [54, 55]. Thus, androgens may have differentially regulated the expression of AR in folliculogenesis in vivo. While PCOS features were induced in female mice by prenatal exposure to nonaromatic DHT, heterozygous and homozygous ARKO mice exposed to DHT maintained comparable ovarian morphology, normal estrous cycling, and corpora lutea numbers [56]; hence, genomic AR signaling could be an important mediator in the development of these PCOS traits. Several ovulation-related genes in the mouse ovary for FSHR, cyclooxyganase-2, and amphiregulin (the epidermal growth factor-like factor) are regulated by nonaromatizable DHT [57, 58]. Ovarian (granulosa) cell-specific ARKO mice showed that AR in GCs is essential to regulate the ovarian gene expression by balancing Ezh2-Jmjd3–mediated H3K27me3 dynamics [42]. However, GABA neuron-specific ARKO mice demonstrated that direct androgen/AR signaling in GABA neurons is largely not required for PCOS-like trait development in the androgenized models of PCOS [59]. Thus, follicle arrest in PCOS may be mainly characterized in GCs by the differential regulation of key genes involved in follicle growth and function. Indeed, our studies revealed that several AR downstream targets, such as *FSHR*, *ANGPT1*, and *LHCGR* genes, which were previously reported to be significantly elevated in patients with PCOS [60–62], were increased in hyperandrogenic ovaries and MPGCs (Fig. 1G, I). Furthermore, the mRNA expression of several FOXL2 downstream genes, such as *CYP19A1*, *FST*, and *STAR* was regulated in hyperandrogenic ovaries and MPGCs under DHT treatment (Fig. 1H, J). Therefore, a complex interplay may exist between androgen/AR signaling and FOXL2 pathways in ovarian cells, particularly affecting genes involved in steroidogenesis (CYP19A1, STAR) and follicular development (FST). Indeed, AR interacted and co-localized with FOXL2 in the nucleus of primary GCs under DHT treatment (Fig. 2) to bind to the promoter II region of the human *CYP19A1* gene to activate its transcription and increase the mRNA expression of *CYP19A1* gene (Fig. 3). The differential mRNA expression of STAR and AR between mouse and human primary granulosa cells may result from variability in follicular stage at the time of sample collection. The primary granulosa cells in our study were collected from 3-week-old C57BL/6 female mice and treated with androgens. In contrast, the human primary granulosa cells from PCOS patients were collected during oocyte retrieval surgery and may be influenced by individual variability and clinical procedures, such as prior exposure to controlled ovarian stimulation during IVF treatment. In addition, laboratory mice typically have homogeneous genetic backgrounds, whereas human PCOS patients exhibit significant genetic heterogeneity and insulin resistance and metabolic dysfunction, which may indirectly affect granulosa cell gene expression. These factors likely contribute to heterogeneous expression patterns not typically observed in animal models, which represent more uniform and controlled conditions. Although KGN cells are known for their ability to produce steroid hormones, mimicking the behavior of granulosa cells and being widely accepted for investigation of PCOS studies as an in vitro granulosa cell model, it should be noted that KGN is a cell line derived from a human ovarian granulosa cell tumor and may not fully reflect the physiological responses of primary granulosa cells. Thus, our results need to be interpreted with caution, and future validation using human primary granulosa cells is necessary to ensure the reliability of our findings. The variability in follicular stage and prior IVF stimulation in human samples may also influence the AR-FOXL2 target genes expression, underlining the need for standardization in sample collection and analysis.

The human *CYP19A1* gene encoded for aromatase has nine coding exons and multiple alternative noncoding first exons used as tissue-specific promoters that contain a distinct set of regulatory sequences controlled by the combined action of specific transcriptional factors in a tissue- and cell-specific manner [25]. The mRNA of *CYP19A1* gene predominantly expressed in GCs and cumulus cells is under the control of PII promoter by several transcriptional regulators, including SF-1, β-catenin, and epigenetic modifications [43, 63, 64]. Recently, *CYP19A1* gene expression was found to be significantly increased, with increased H3K9ac (activation marker) and decreased H3K9me2 (repression markers), at the PII promoter of *CYP19A1* gene in cumulus cells in PCOS [64]. The insight into whether these epigenetic mechanisms are involved in the AR-FOXL2 to cooperatively mediate *CYP19A1* gene transcription in patients with PCOS remains unclear.

The forkhead box (FOX) protein family comprises evolutionarily conserved transcription factors that act as pioneering factors to establish gene expression patterns and control biological processes [65]. FOXA1 and FOXA2, members of this family, are known to interact with AR to regulate transcriptional programs in prostate cancer [66]. Since we found that AR interacts with FOXL2 to induce CYP19A1 gene transcription in GCs, it would be interesting to determine whether AR signaling collaborates with other FOX proteins to activate CYP19A1 gene transcription and control estrogen production in other tissues (e.g., testis, prostate, adipose tissue, or placenta) where aromatase is widely expressed.

Several FOXL2 downstream genes associated with folliculogenesis and steroidogenesis were distinctly expressed in the ovaries of PCOS mice (Fig. S2). For instance, FOXL2 directly modulates functional enhancer region of ESR2 gene in GCs [67] and the peaks of FOXL2 ChIP in mouse ovaries were highly enriched in steroid receptor motifs such as ESR1/2 and AR [68]. The FSHR gene, which has also been shown to be modulated by FOXL2, plays a crucial role in mediating FSH actions that are enhanced by androgen/AR signaling [58, 62, 69]. Given this dual regulation, it would be valuable to conduct ChIP-seq analysis to identify downstream genes involved in aberrant PCOS folliculogenesis that might be cooperatively modulated by FOXL2 and androgen/AR signaling. Our findings demonstrate that AR-FOXL2 complex formation is androgen-dependent, suggesting that PCOS patients with different degrees of hyperandrogenism may exhibit varying levels of this protein complex interaction. Therefore, variations in AR sensitivity or FOXL2 expression may result in different magnitudes of CYP19A1 activation, leading to variable estrogen production among patients. This mechanism may partially explain why some PCOS patients have relatively normal estrogen levels while others exhibit significant elevation. Together, variations in the expression levels or functional activity of the overlapping downstream gene network modulated by FOXL2 and AR may contribute to the PCOS heterogeneity and phenotypic diversity.

Furthermore, the AR-FOXL2 signaling status could be assessed through minimally invasive procedures. Granulosa cells obtained during routine follicular aspiration for IVF could be analyzed for AR and FOXL2 expression patterns, providing personalized treatment guidance without additional patient burden. In addition, the development of non-invasive biomarkers based on this pathway could be valuable. Circulating levels of CYP19A1 protein, or specific metabolites reflecting AR-FOXL2 activity, may potentially be measured in blood samples to stratify patients and monitor treatment responses.

Our findings on the AR-FOXL2-aromatase axis in PCOS provide crucial mechanistic insights that strengthen the clinical evidence supporting the use of letrozole for ovulation induction. By demonstrating that hyperandrogenism leads to increased estrogen synthesis through AR-FOXL2-mediated activation of the aromatase gene in GCs, our study explains why the targeted inhibition of aromatase by letrozole is particularly effective in PCOS patients [70]. Mechanistically, aromatase inhibitors block the conversion of androgens to estrogens in ovarian follicles, resulting in decreased circulating estrogens. The reduction in estrogen levels releases the hypothalamo-pituitary axis from estrogen’s negative feedback, triggering a surge in FSH which promotes normal follicular development with selection of a dominant follicle and atresia of smaller follicles, thereby facilitating follicular growth and ovulation.

This molecular understanding aligns with clinical trial data showing the superiority of letrozole compared to clomiphene citrate in PCOS patients, with higher ovulation rates (82.4% vs. 63.6%), better pregnancy rates (21.6% vs. 9.1%), and lower multiple pregnancy rates (0% vs. 3.2%) [71]. Furthermore, by revealing the role of the AR-FOXL2-aromatase axis in granulosa cell estrogen synthesis, our findings not only validate these clinical observations but also provide potential new therapeutic insights for targeting this pathway, establishing a strong scientific foundation for letrozole as a treatment option for ovulation induction in PCOS patients.

During controlled ovarian hyperstimulation cycles, the GCs in PCOS display an exaggerated response to exogenous FSH, thereby augmenting estrogen production. However, our data showed that the MPGCs, the mouse follicles, and the KGN cell line were cultured with androgens stimulation in vitro, they exhibit substantially increased *CYP19A1* gene expression (Fig. 1J, S4B and 3A) and estrogen production (Fig. 4D, S5). These data suggest that in an isolated environment, androgen stimulation acts directly on the GCs to increase CYP19A1 expression and estrogen production without controlled ovarian hyperstimulation as a potential confounding factor.

## Conclusion

Due to the heterogeneity of excess androgens and multiple hormonal imbalances in PCOS, the severity and combination of symptoms can vary widely among individuals, leading to different PCOS phenotypes. By combining clinical observations with targeted in vivo and in vitro models, this study revealed androgen impact ovarian function, gene expression, and hormonal production, contributing to the complex pathophysiology of PCOS. Notably, we demonstrate that AR directly interacts with FOXL2 to regulate CYP19A1 gene expression in GCs, thereby modulating increased estrogen synthesis in PCOS. This study may provide valuable insights into the role of chronically elevated estrogens in contributing to the characteristic features of PCOS, including persistent hormonal imbalances, anovulation, metabolic disturbances such as insulin resistance, dyslipidemia, and central adiposity. Moreover, it may also offer a potential explanation for these diverse clinical presentations observed in PCOS, highlighting the complex interplay between ovarian steroidogenesis and systemic metabolic regulation. Nonetheless, it is important to acknowledge that this study is primarily focused on granulosa cells from PCOS patients with hyperandrogenism undergoing IVF treatment, which may not fully represent the complete spectrum of PCOS phenotypes. This introduces several important limitations. Whether similar molecular mechanisms operate in non-IVF populations or in PCOS variants with distinct metabolic features remains unclear. PCOS patients with significant insulin resistance may have altered granulosa cell function due to hyperinsulinemia’s effects on steroidogenesis, which could modify AR-FOXL2 interactions independently of androgen levels. Our study’s focus on predominantly reproductive aspects may not capture the full complexity of PCOS pathophysiology in metabolically distinct phenotypes. These considerations underscore the need for caution when generalizing our results to the entire PCOS population. Future investigation of AR-FOXL2-mediated signaling across a more diverse range of clinical phenotypes, including non-IVF and metabolically heterogeneous PCOS patients, is warranted to strengthen clinical relevance. Given that PCOS is a multifactorial condition involving insulin resistance, inflammation, and neuroendocrine dysregulation, our findings represent only one component of this complex disease. Further studies are needed to elucidate the AR-FOXL2 interaction and its downstream transcriptional effects.

## Supplementary Information

Below is the link to the electronic supplementary material.

Supplementary Material 1

Supplementary Material 2

Supplementary Material 3

Supplementary Material 4

Supplementary Material 5

Supplementary Material 6

Supplementary Material 7

Supplementary Material 8

Supplementary Material 9

Supplementary Material 10

Supplementary Material 11

Supplementary Material 12

Supplementary Material 13

Supplementary Material 14

Supplementary Material 15

Supplementary Material 16

Supplementary Material 17

Supplementary Material 18

Supplementary Material 19

Supplementary Material 20

Supplementary Material 21

Supplementary Material 22

Supplementary Material 23

Supplementary Material 24

Supplementary Material 25

Supplementary Material 26

Supplementary Material 27

Supplementary Material 28

Supplementary Material 29

Supplementary Material 30

Supplementary Material 31

Supplementary Material 32

Supplementary Material 33

Supplementary Material 34

Supplementary Material 35

Supplementary Material 36

## Data Availability

No datasets were generated or analysed during the current study.

## Abbreviations

PCOS: Polycystic ovary syndrome
AR: Androgen receptor
FOXL2: Forkhead box protein L2
DHT: Dihydrotestosterone
LH: Luteinizing hormone
GCs: Granulosa cells
MPGCs: Mouse primary granulosa cells
E1: Estrone
E2: 17β-Estradiol
CYP19A1: Cytochrome P450 family 19 subfamily A member 1
FSH: Follicle-stimulating hormone
ARKO: AR-knockout
IVF: In vitro fertilization
ELISA: Enzyme-linked immunosorbent assay
IHC: Immunohistochemical
IF: Immunofluorescence
scRNA-seq: Single-cell RNA sequencing
LPAFs: Large preantral follicles
IP: immunoprecipitation
ChIP: Chromatin immunoprecipitation
FSHR: FSH receptor
VE: Vehicle
ANGPT1: Angiopoietin 1
LHCGR: LH/choriogonadotropin receptor
FST: Follistatin
STAR: Steroidogenic acute regulatory protein
ESR: estrogen receptor
