# Supplementary material for: Hyperandrogenism in polycystic ovary syndrome augments Estrogen synthesis through AR-FOXL2–mediated activation of the aromatase gene in granulosa cells
Source: J Ovarian Res. 2025 Sep 2;18:200. doi: 10.1186/s13048-025-01790-4 (PMC12406384; doi:10.1186/s13048-025-01790-4)
Supplement: Supplementary file 1 — Supplementary Material 1 [file 13048_2025_1790_MOESM1_ESM.pdf]

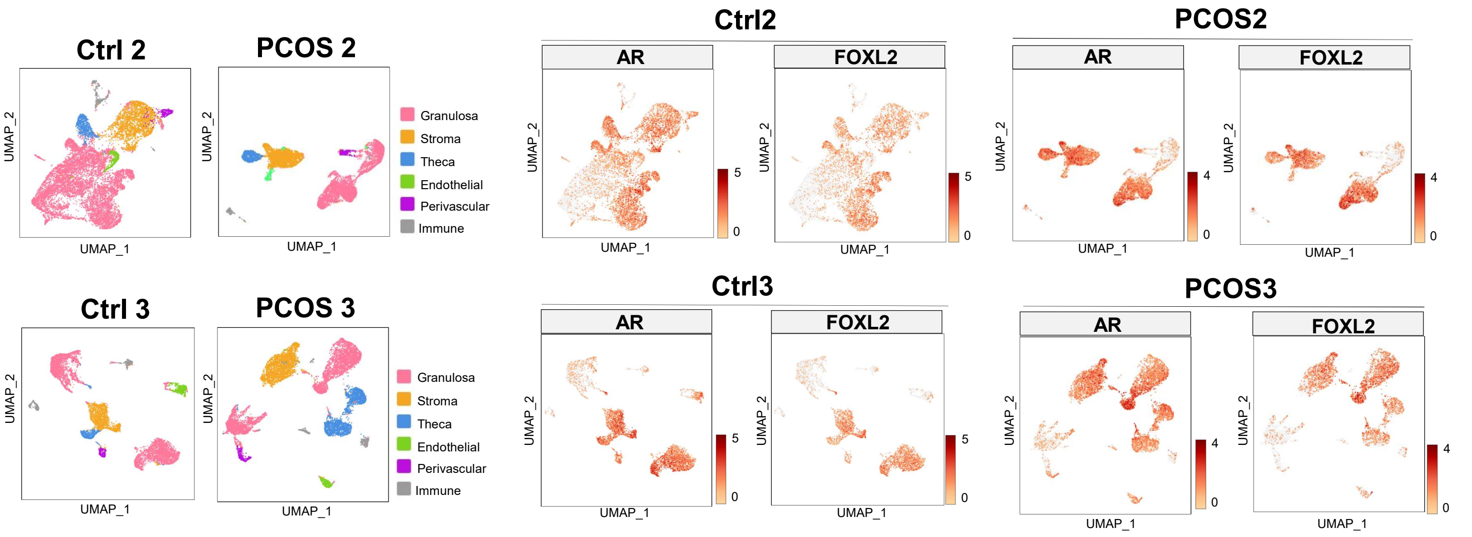

Supplementary Figure 1. UMAP plots and feature plots illustrating the similar distribution of AR and FOXL2 within ovarian cell populations from two additional sets of control and PCOS mice.

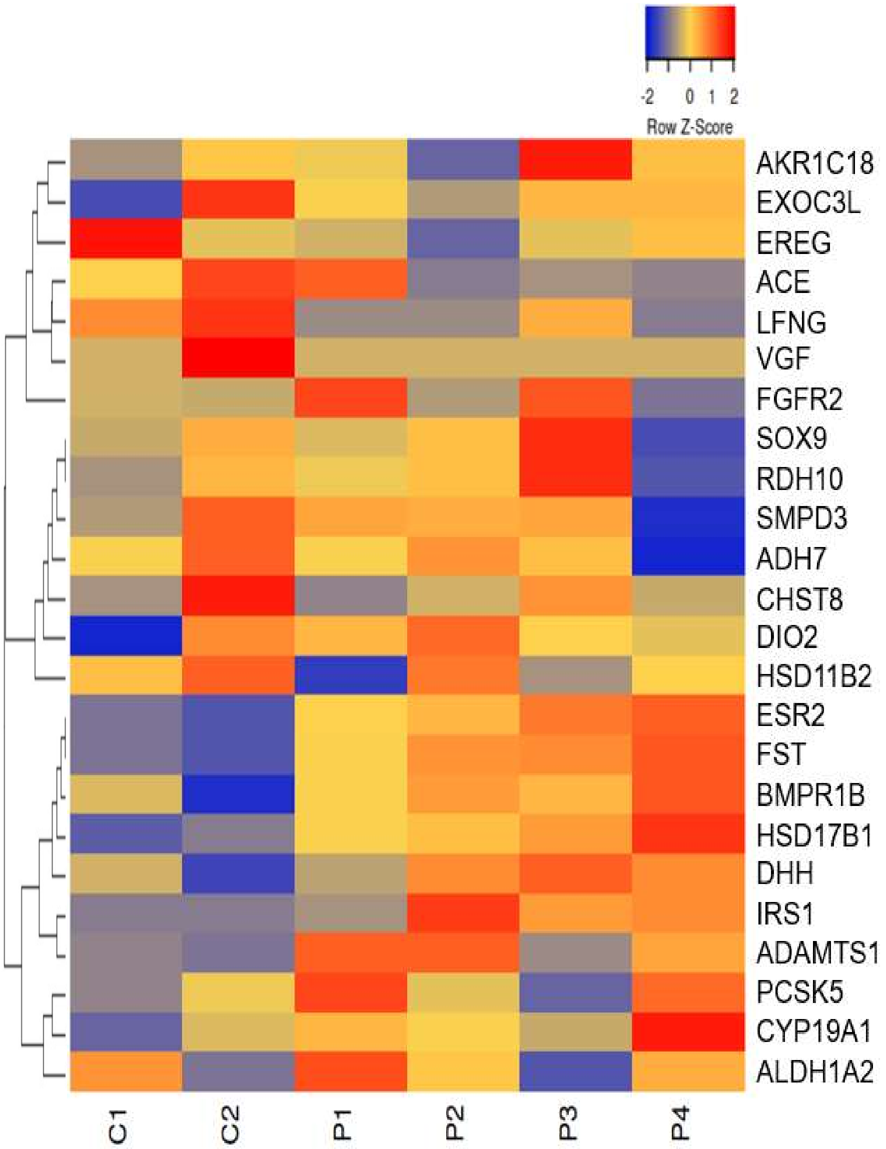

Supplementary Figure 2. By using a public dataset (GSE148839) containing ovaries from PCOS mice and control mice, a hierarchical clustering of FOXL2 downstream target genes related to the regulation of hormone levels and reproductive structure development was identified .

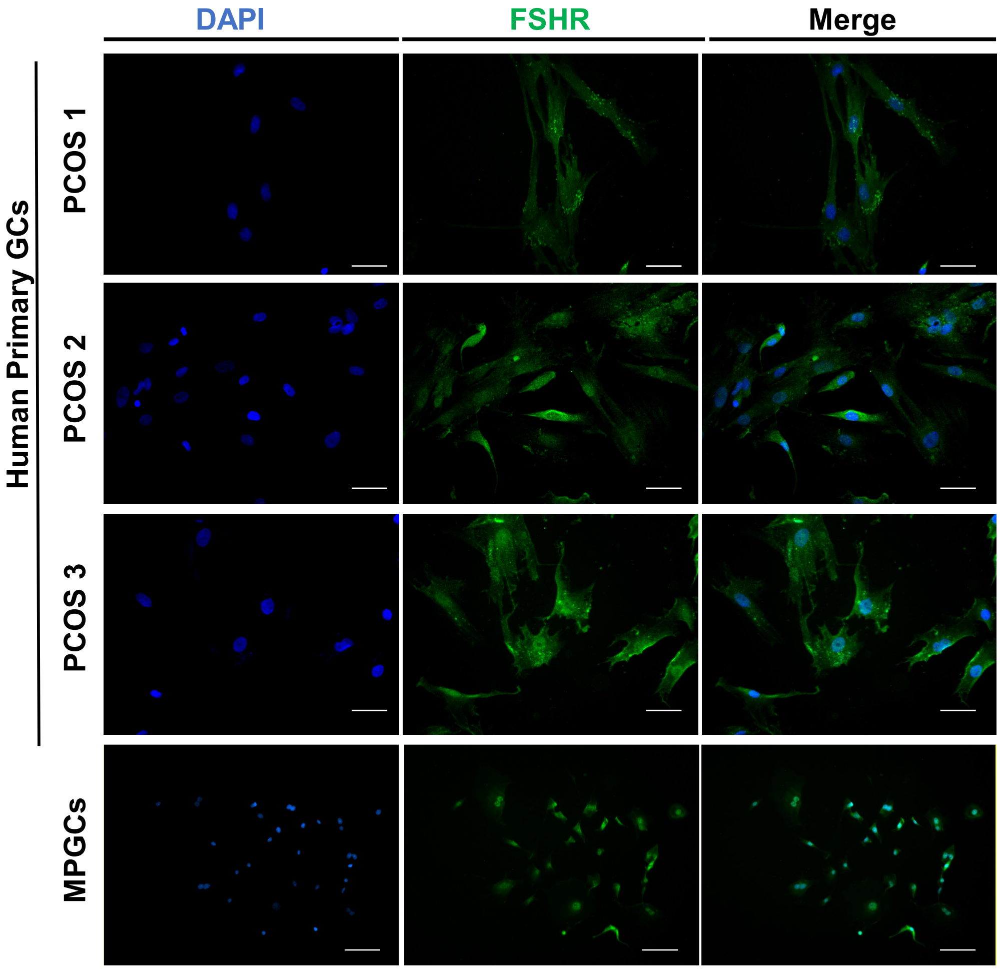

Supplementary Figure 3. Expression of FSH receptor (FSHR), a granulosa cell marker, was verified by immunofluorescence (IF) staining in primary GCs from PCOS patients and in mouse primary granulosa cells (MPGCs) isolated from 3-week-old mice. Bars represent 100 μm.

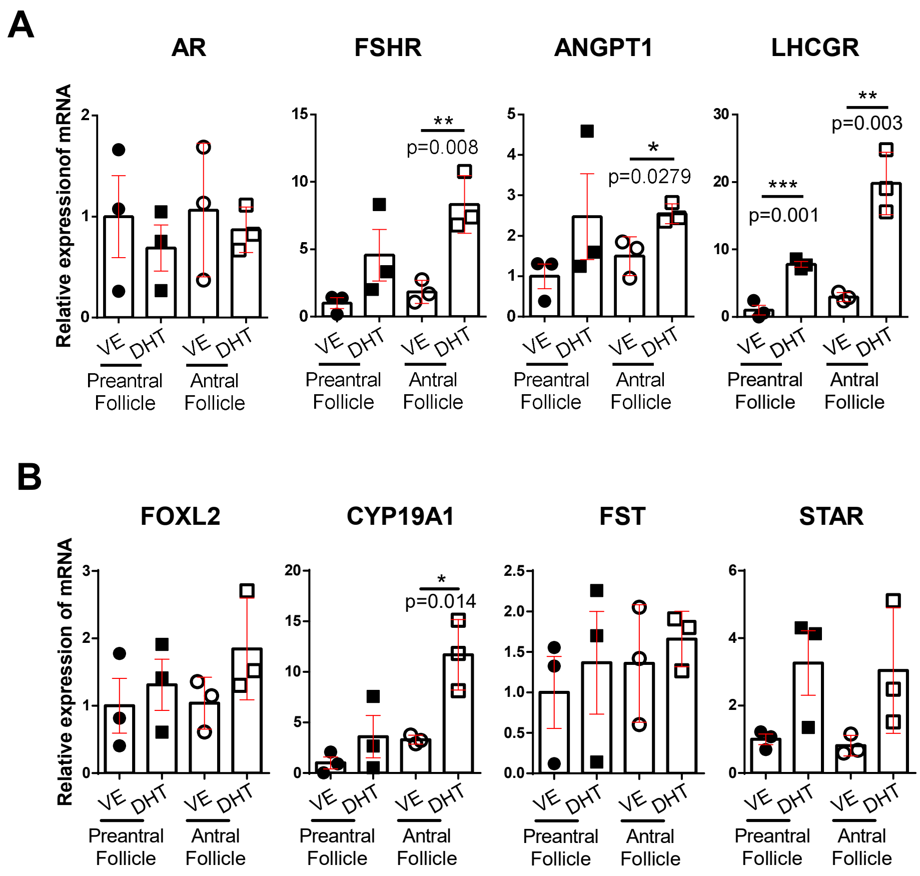

Supplementary Figure 4. The relative mRNA expression of AR, FOXL2 and their downstream target genes in follicles under DHT treatment. Expression change of (A) AR and its downstream genes, and (B) FOXL2 and its downstream genes in 5 pooling preantral follicles or 10 pooling antral follicles from 6-day *in vitro* culture with or without 1 μM DHT (n=3). Data were analyzed by one-way ANOVA and are expressed as the mean ± SEM; *, $p<0.05$; **, $p<0.01$; ***, $p<0.001$.

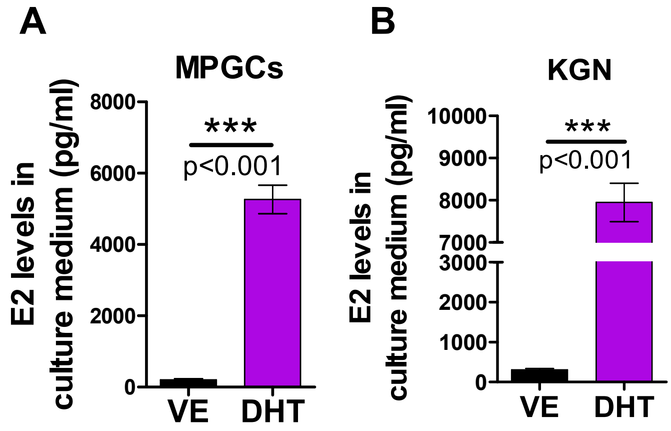

Supplementary Figure 5. Androgen excess increased estrogen levels in granulosa cells. The levels of E2 in (A) MPGCs, (B) KGN culture medium were tested by ELISA after DHT treatments for 48 hours (n=3).

Supplementary table 1. Primers list

Gene	**Forward Sequences (5'-3')**	**Reverse Sequences (5'-3')**
Mouse		
β-actin	AGGCCAACCGTGAAAAGATG	TGTGGTACGACCAGAGGCATAC
FOXL2	GGACAGCTTCTGGATGCAGAGCC	CAGCGGAGGCGCAAAGCGGAGTCGCAGG
CYP19A1	GAGAGTTCATGAGAGTCTGGATCA	CATGGAACATGCTTGAGGACT
FST	CTCTCTCTGCGATGAGCTGTGT	TCTTCCTCCTCCTCCTCTTCCT
STAR	CCGGAGCAGAGTGGTGTCA	CAGTGGATGAAGCACCATGC
AR	GCTGCCTTGTTATCTAGCCTCAA	AATGACCGCCATCTGGTCAT
FSHR	CCTTGCTCCTGGTCTCCTTG	CTCGGTCACCTTGCTATCTTG
ANGPT1	GGGGGAGGTTGGACAGTAA	CATCAGCTCAATCCTCAGC
LHCGR	GATGCACAGTGGCACCTTC	TCAGCGTGGCAACCAGTAG
Human		
β-actin	TCACCCACACTGTGCCCATCTACGA	CAGCGGAACCGCTCATTGCCAATGG
FOXL2	ATCTACCAGTACATCATCGC	GCCCTTCTTATTCTTCTCGT
CYP19A1	TGCCACCATGCCAGTCCTGC	TGCCGTGGGAGATGAGGGGT
FST	TGCTCTGCCAGTTCATGG	CTTGACGGAGCCAGCAGT
STAR	GGCTACTCAGCATCGACCTC	CATCCCACTGTCACCAGATG
AR	TCACCGCACCTGATGTGTG	ACATGGTCCCTGGCAGTCTC
CYP19A1 promoter AREⅠ	TGGAAGGCTCTGAGAAGACC	GCAATCTCCCAACTCCCCTT
CYP19A1 promoter AREⅡ	TGCCTAAACAAAACCTGCTGA	TGGGTAGAGTGACGTGCATT
CYP19A1 promoter AREⅢ	TGGGCTTCCTTGTTTTGACT	TGTTGCTTCAGAGGGTGCT

Supplementary table 2. Antibodies list

Antibody	Catalog	Company	Identifier	Dilution factor				
				IHC	IF	WB	IP	ChIP
AR	ab133273	Abcam	RRID:AB_11156085	500		1000		
AR	06-680	Millipore	RRID:AB_310214				100	
AR	sc-7305	Santa cruz	RRID:AB_626671		200			
AR	5153S	Cell signaling	RRID:AB_10691711					100
FOXL2	ab246511	Abcam	RRID:AB_2895602	8000	100	1000	200	
Aromatase	GTX18995	Genetex	RRID:AB_510151	100				
FSHR	22665-1-AP	Proteintech	RRID:AB_2631204		100			
GAPDH	MA5-15738	Invitrogen	RRID:AB_10977387			10000		
Normal rabbit IgG	sc-2027	Santa cruz	RRID:AB_737197				150	
