# Supplementary material for: Hyperandrogenism in polycystic ovary syndrome augments Estrogen synthesis through AR-FOXL2–mediated activation of the aromatase gene in granulosa cells
Source: J Ovarian Res. 2025 Sep 2;18:200. doi: 10.1186/s13048-025-01790-4 (PMC12406384; doi:10.1186/s13048-025-01790-4)
Supplement: Supplementary file 36 — Supplementary Material 36 [file 13048_2025_1790_MOESM36_ESM.pdf]

Fig. 2E

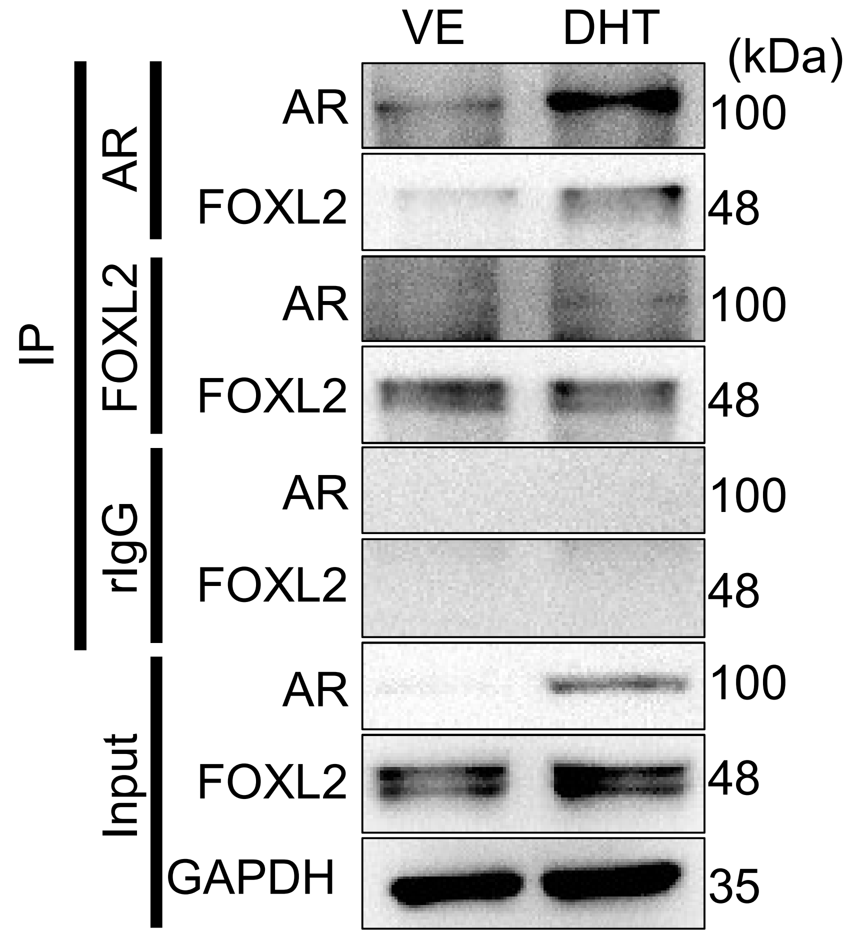

AccuRuller RGB Prestained Protein Ladders

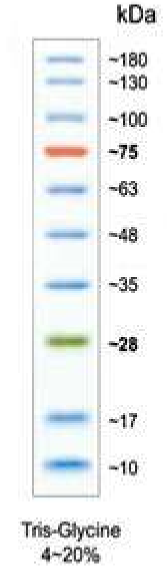

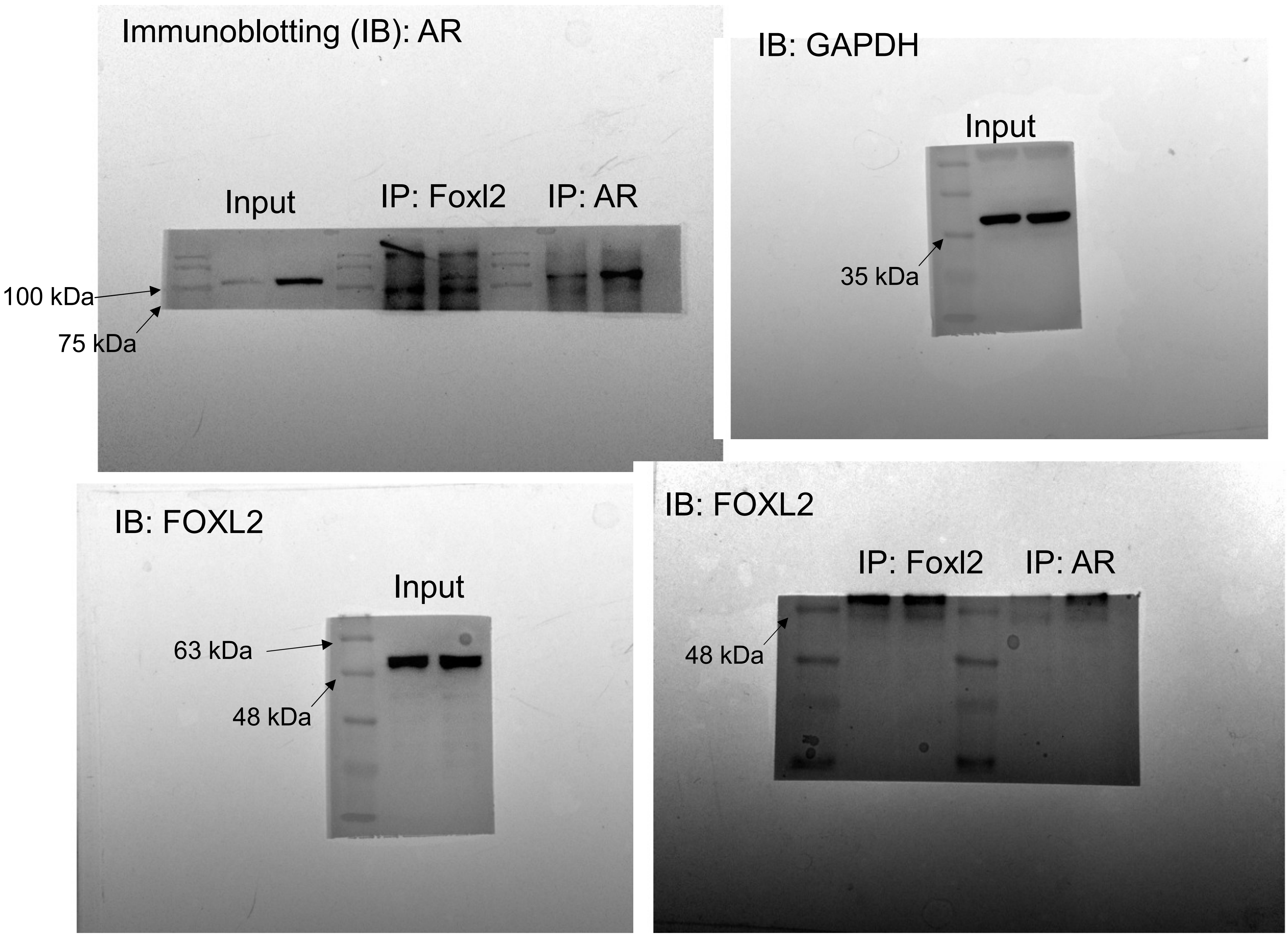
Immunoblotting (IB): AR
Input
IP: Foxl2
IP: AR
100 kDa
75 kDa
IB: GAPDH
Input
35 kDa
IB: FOXL2
Input
63 kDa
48 kDa
IB: FOXL2
IP: Foxl2
IP: AR
48 kDa

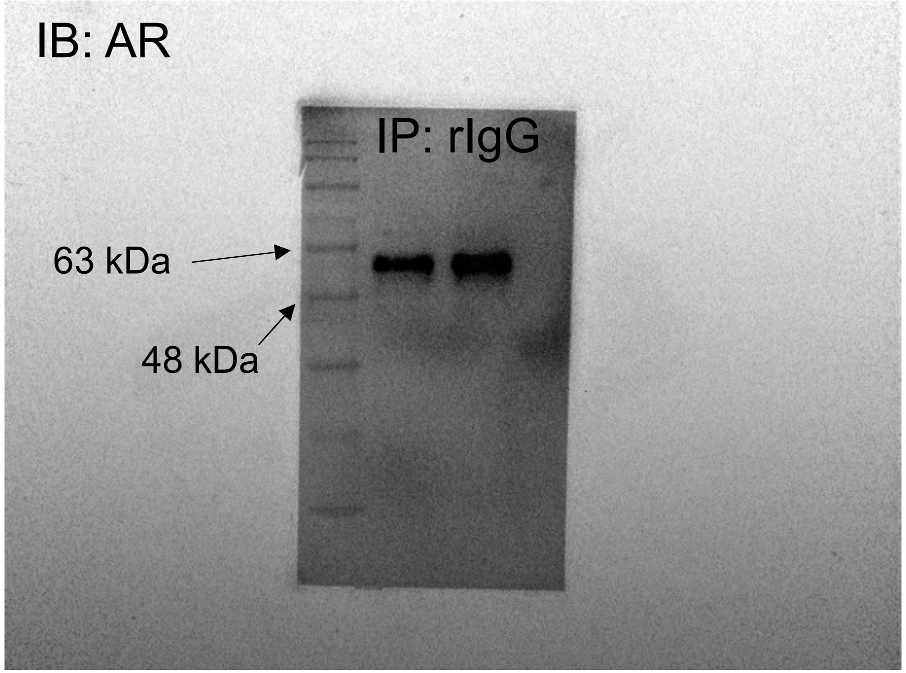
IB: AR
IP: rIgG
63 kDa
48 kDa

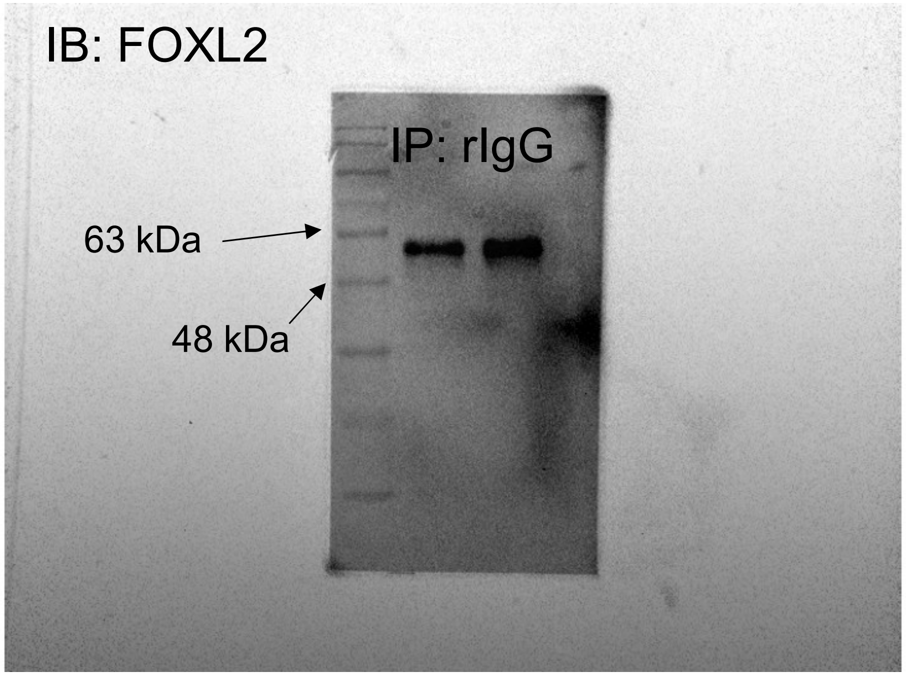
IB: FOXL2
IP: rIgG
63 kDa
48 kDa

Fig. 3E

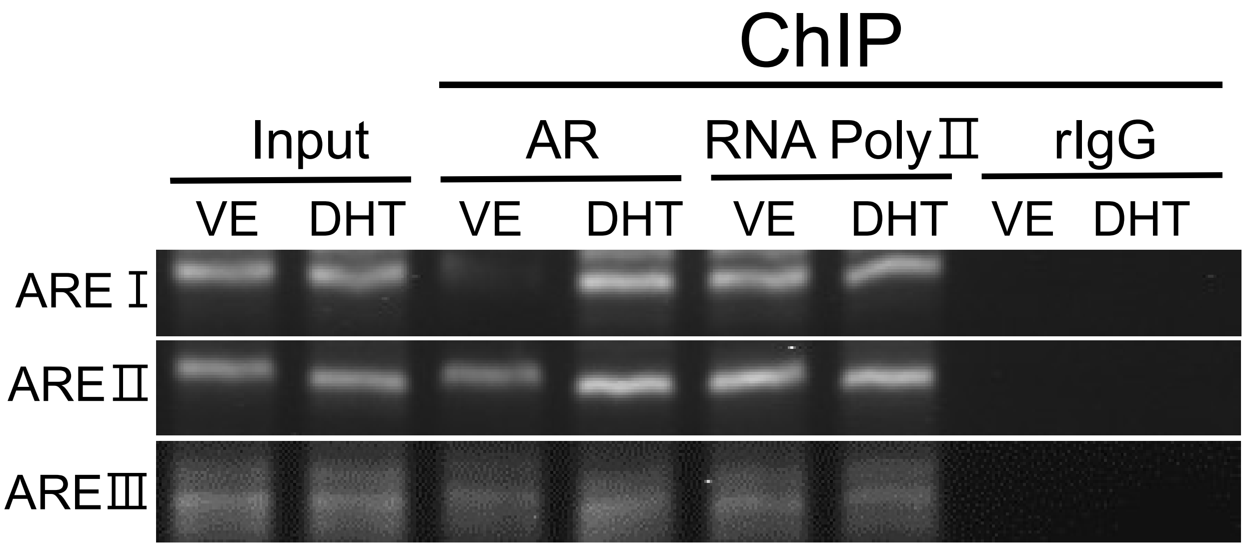

ChIP
Input
AR
RNA PolyⅡ
rIgG
VE
DHT
VE
DHT
VE
DHT
VE
DHT
AREⅠ
AREⅡ
AREⅢ

Bio-100 Mass DNA Ladder, PT-M1-100T

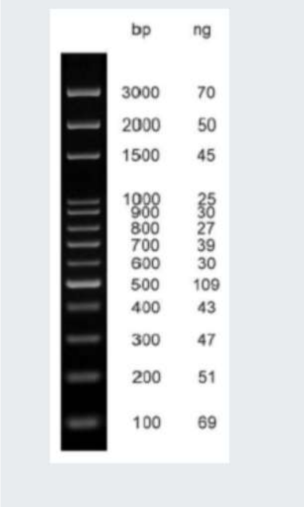

2% Agarose Gel in 0.5X TBE Buffer(Amresco, Agarose I)

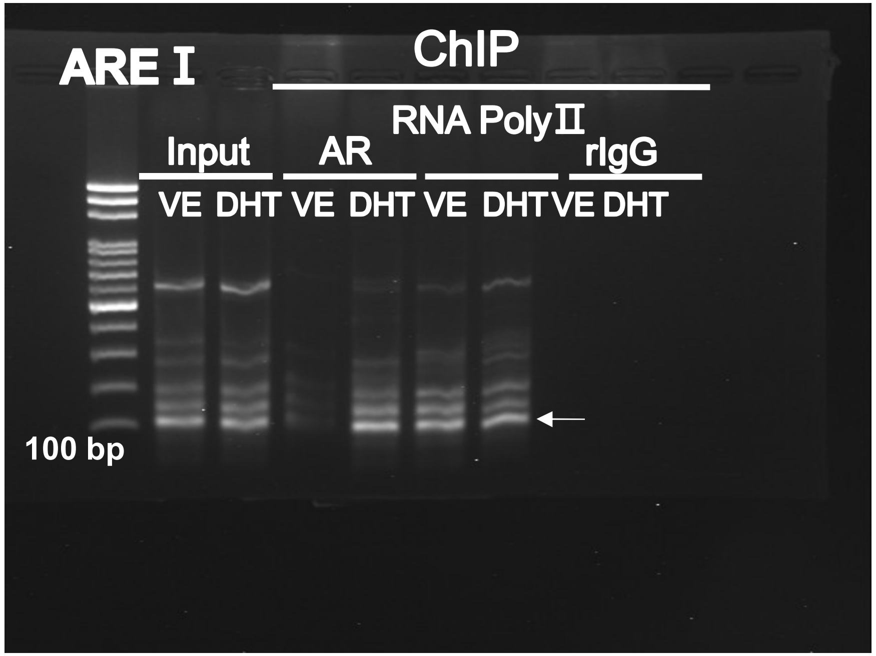

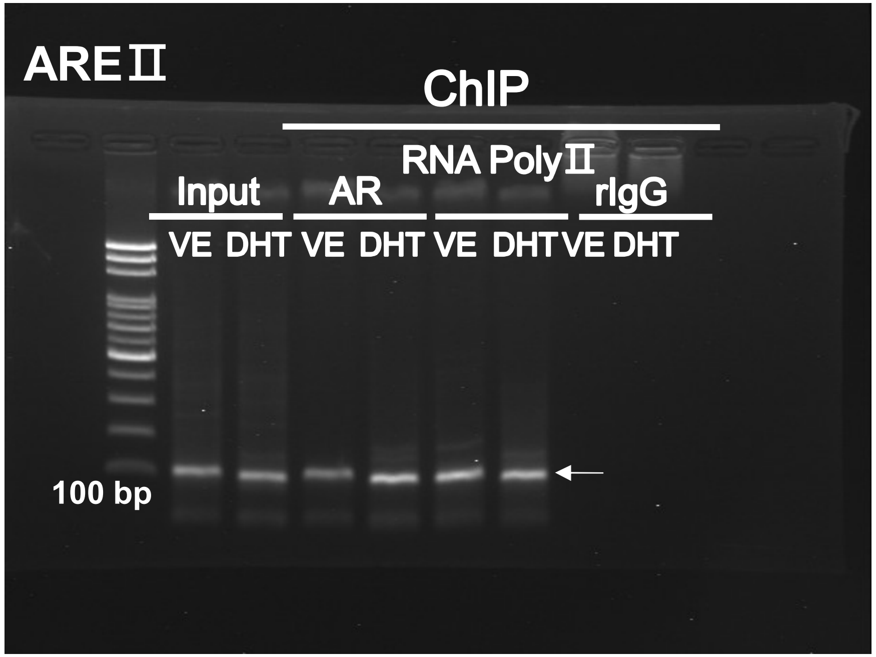

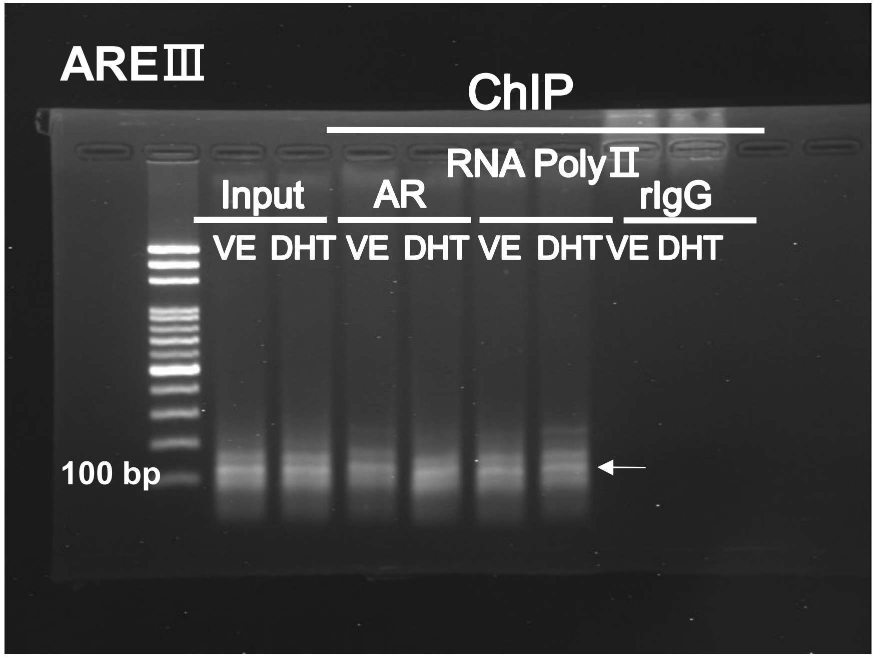

Fig. 3G

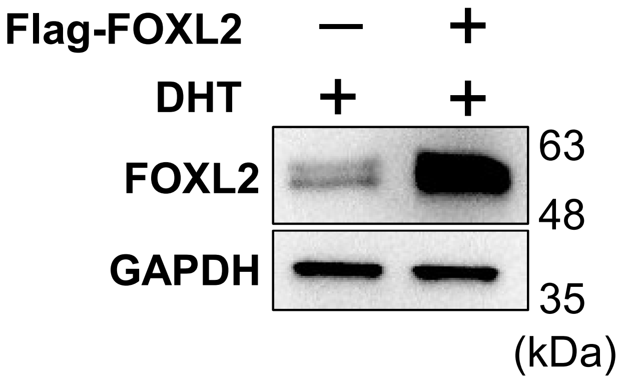
Flag-FOXL2
—
+
DHT
+
+
FOXL2
GAPDH
63
48
35
(kDa)

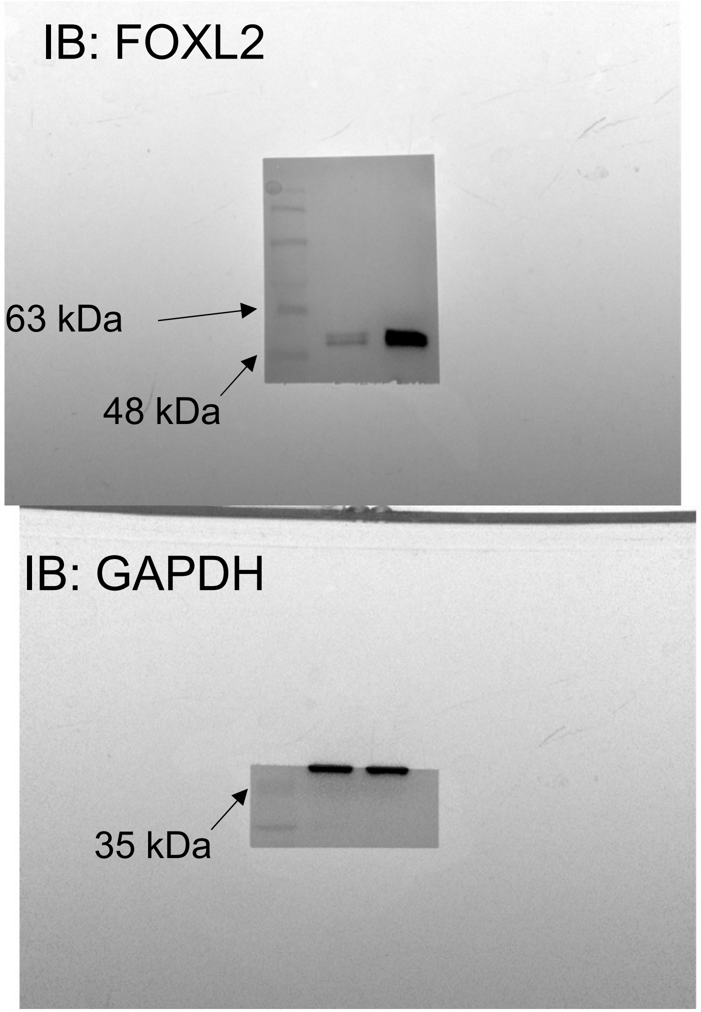
IB: FOXL2
63 kDa
48 kDa
IB: GAPDH
35 kDa

Fig. 3H

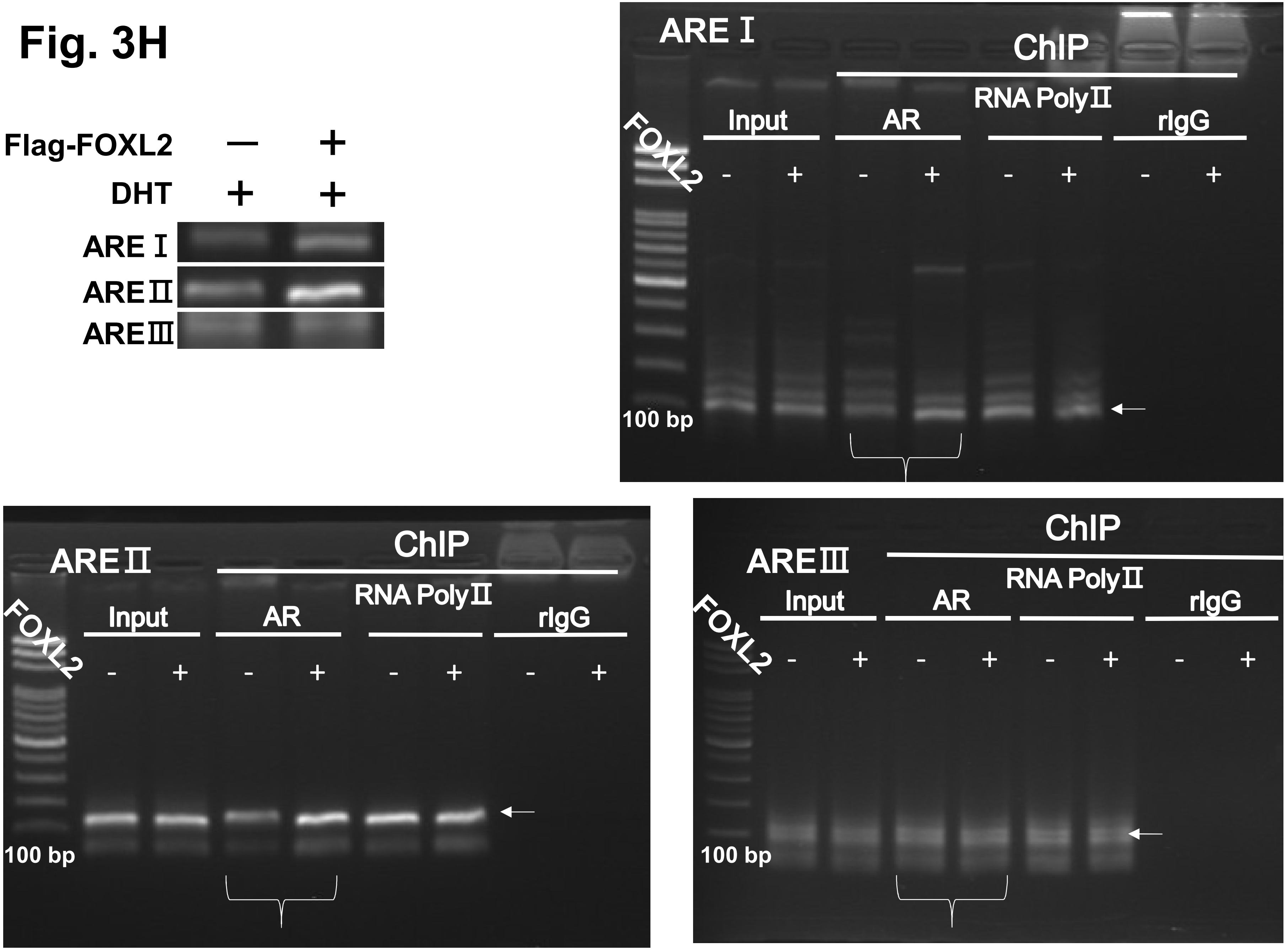
Flag-FOXL2 — +
DHT + +
ARE Ⅰ
ARE Ⅱ
ARE Ⅲ
ARE Ⅰ
ChIP
FOXL2
Input
AR
RNA Poly Ⅱ
rIgG
- + - + - + - +
100 bp
ARE Ⅱ
ChIP
FOXL2
Input
AR
RNA Poly Ⅱ
rIgG
- + - + - + - +
100 bp
ARE Ⅲ
ChIP
FOXL2
Input
AR
RNA Poly Ⅱ
rIgG
- + - + - + - +
100 bp

Fig. 3J

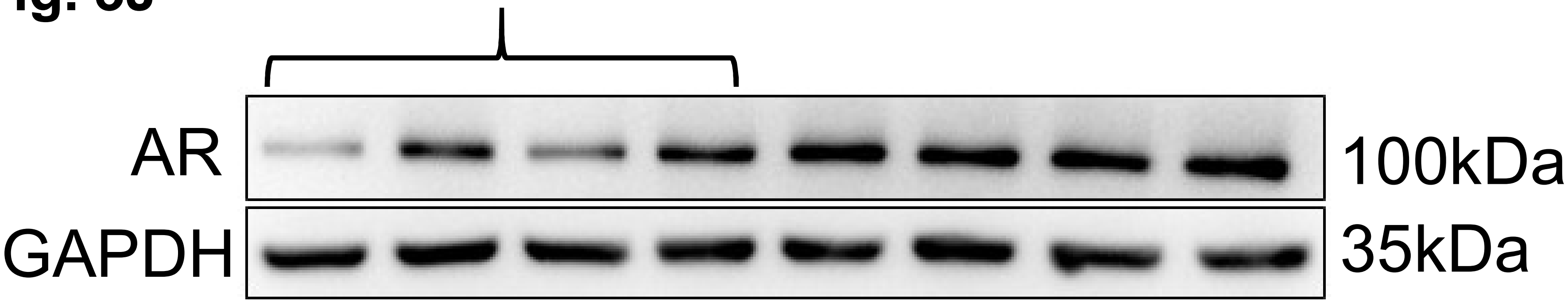
AR
100kDa
GAPDH
35kDa

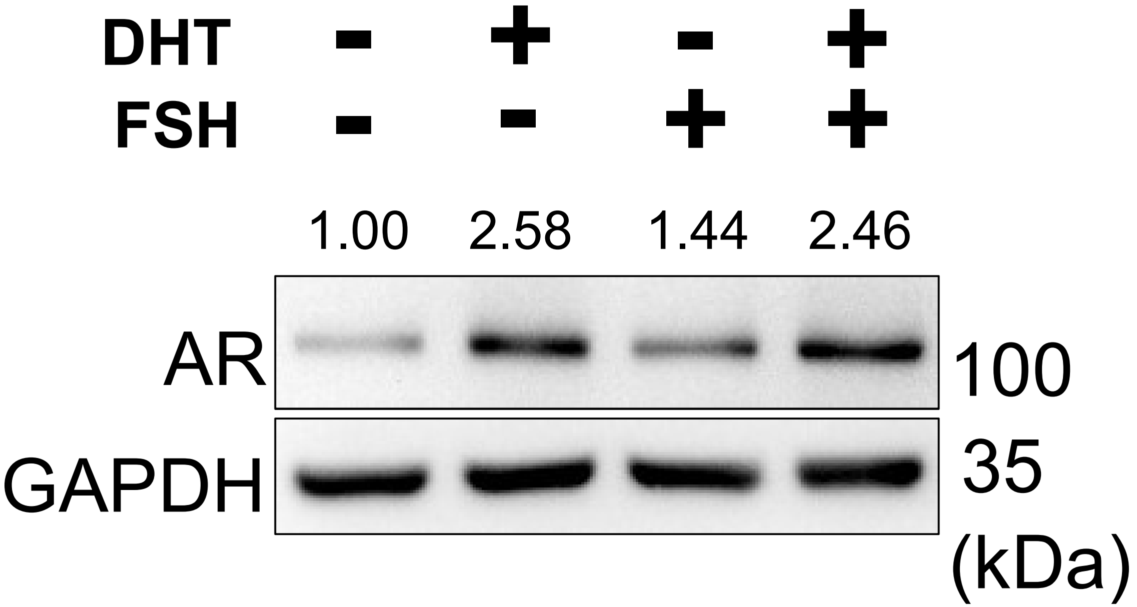
DHT - + - +
FSH - - + +
1.00 2.58 1.44 2.46
AR
100
GAPDH
35
(kDa)

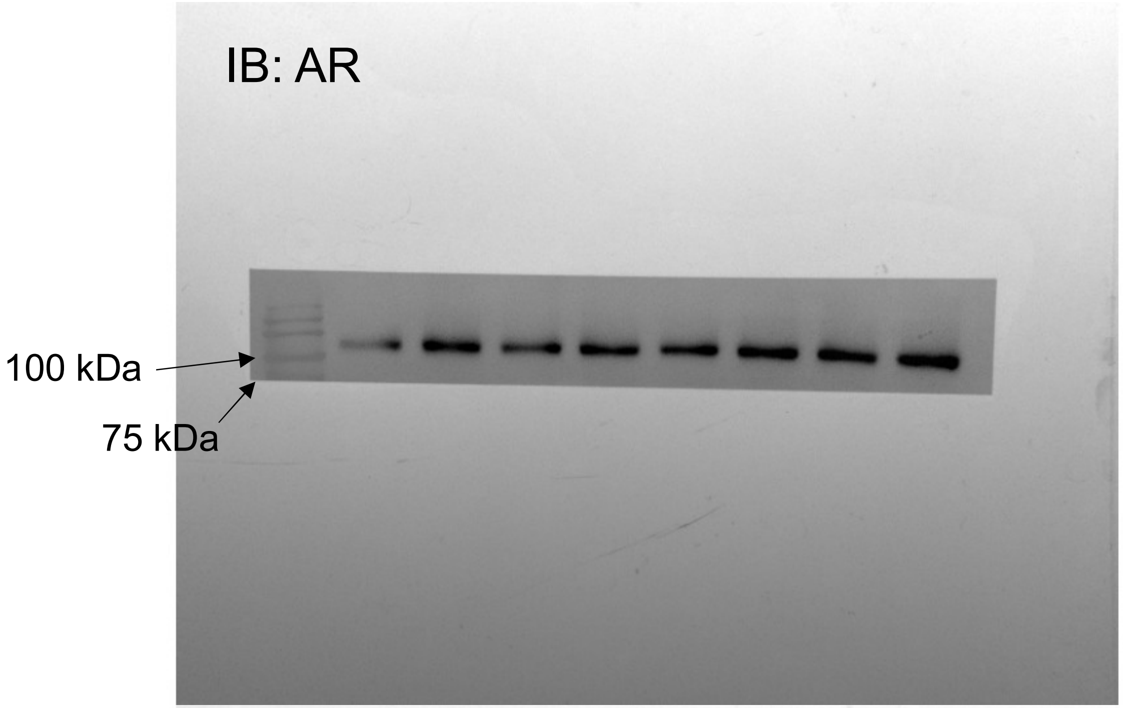
IB: AR
100 kDa
75 kDa

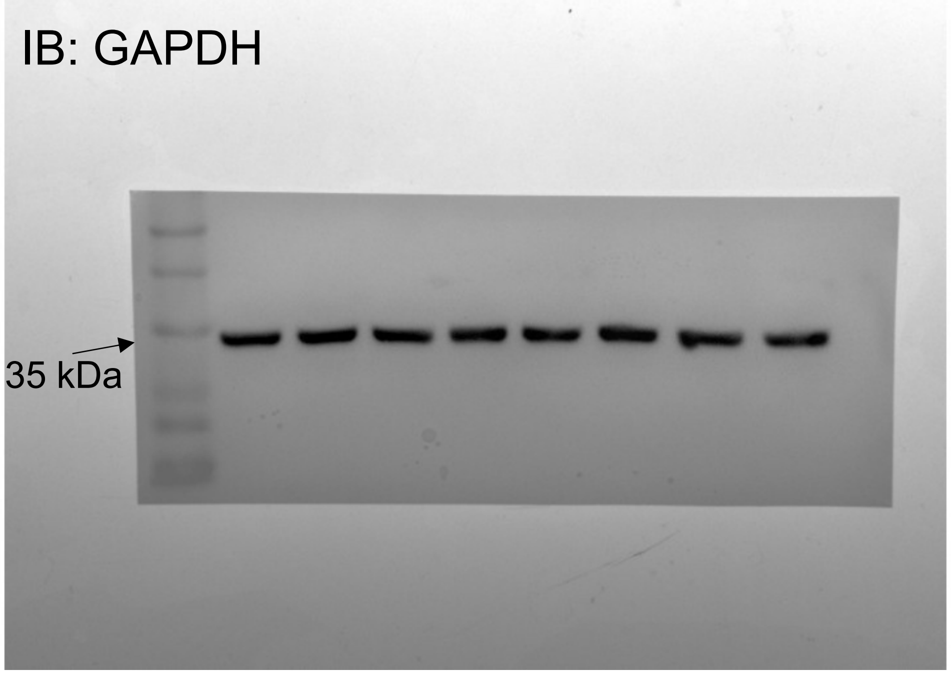
IB: GAPDH
35 kDa

Fig. 3K

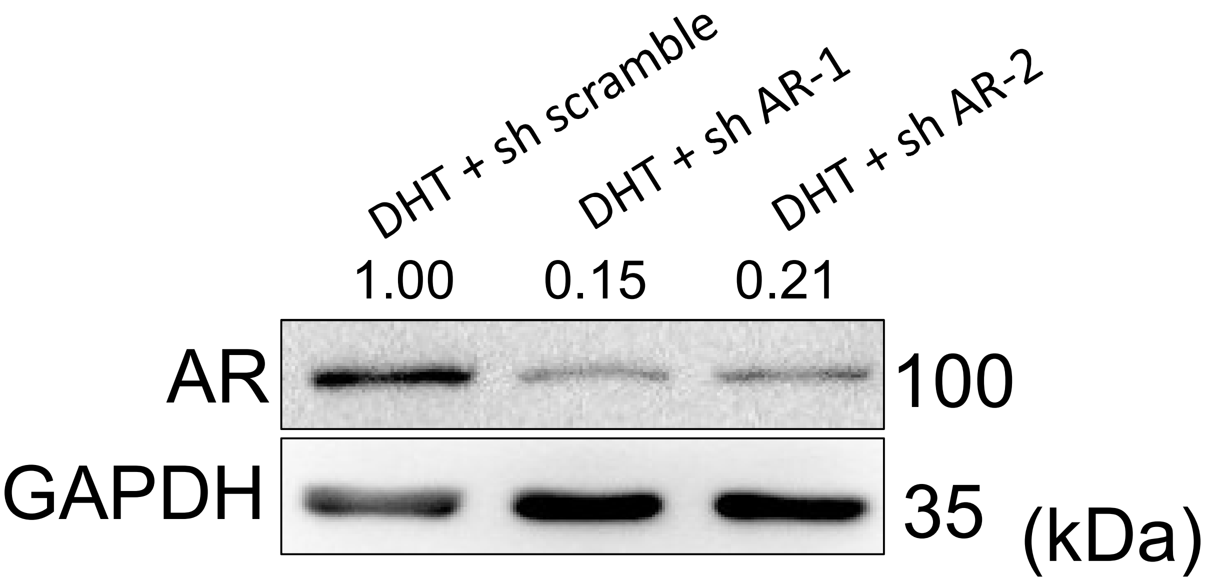
DHT + sh scramble
DHT + sh AR-1
DHT + sh AR-2
1.00
0.15
0.21
AR
100
GAPDH
35
(kDa)

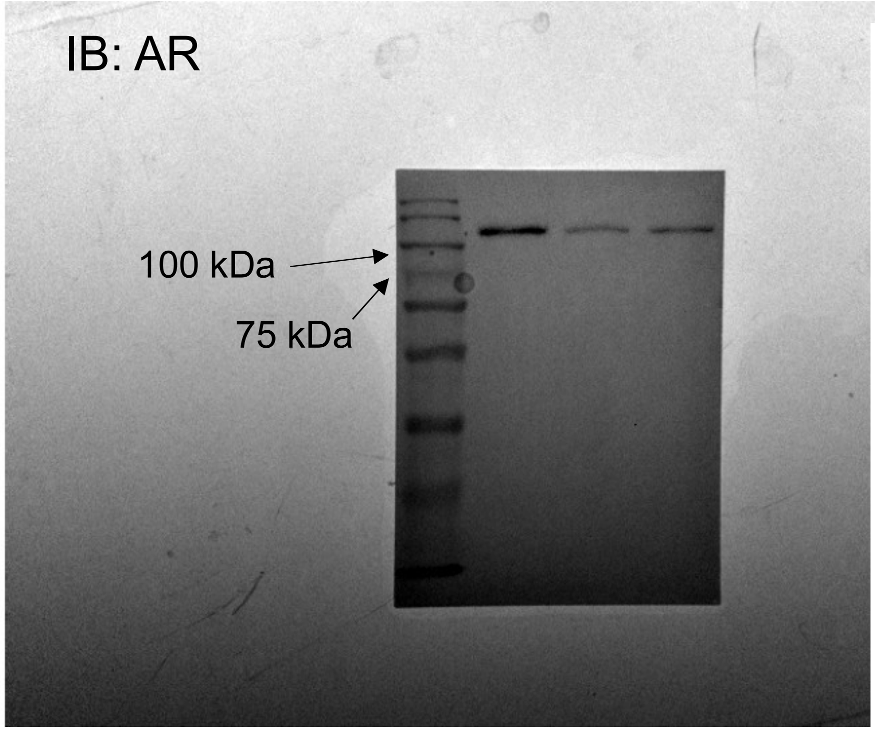
IB: AR
100 kDa
75 kDa

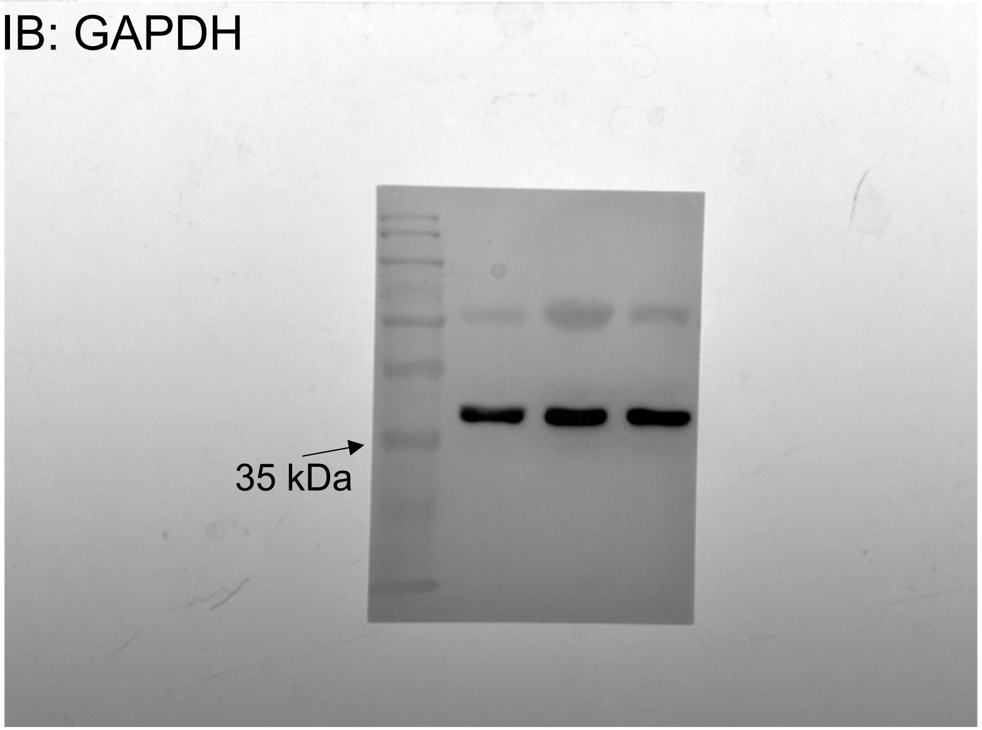
IB: GAPDH
35 kDa

Fig. 4E

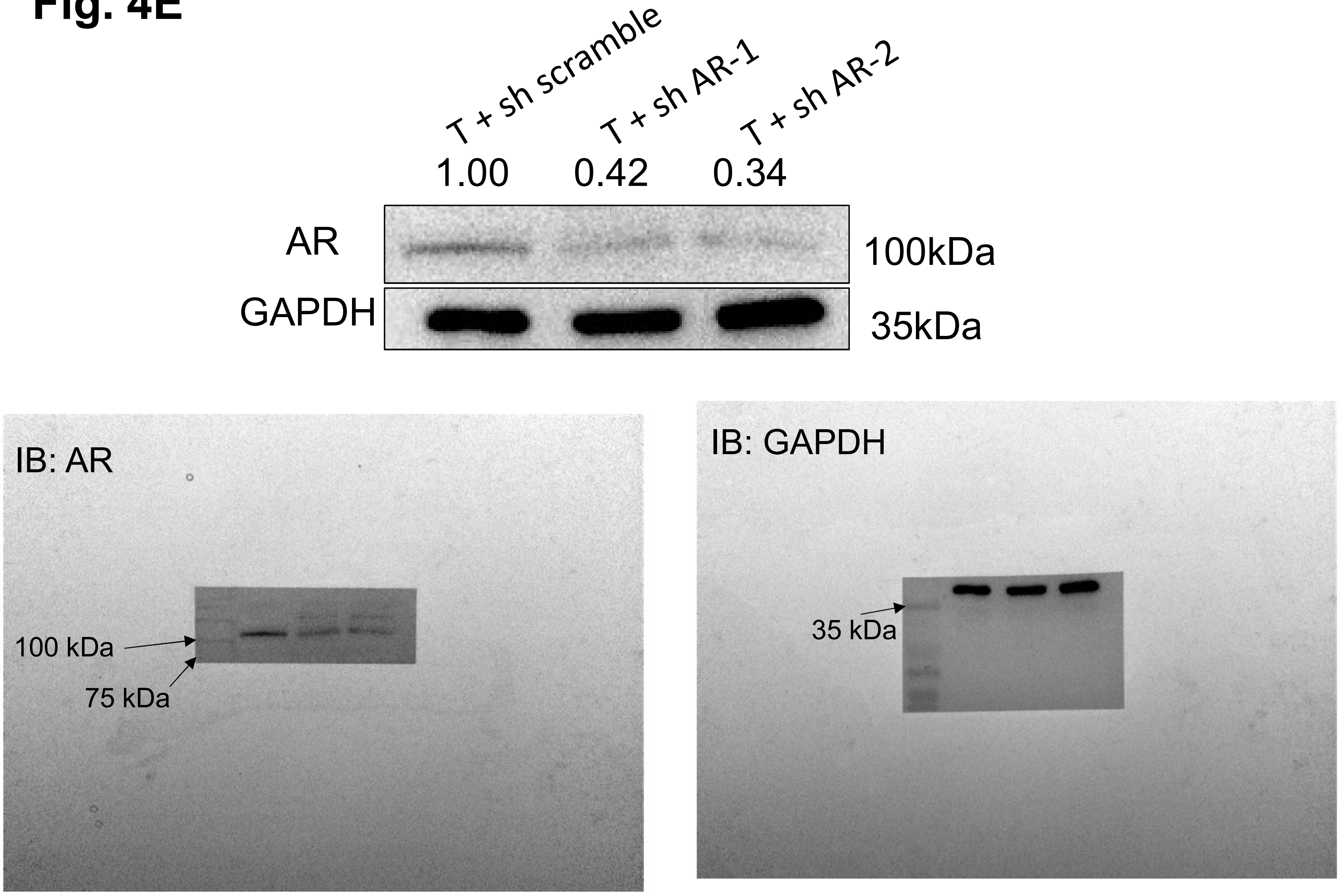
T + sh scramble
T + sh AR-1
T + sh AR-2
1.00
0.42
0.34
AR
100kDa
GAPDH
35kDa
IB: AR
100 kDa
75 kDa
IB: GAPDH
35 kDa

Fig. 4F

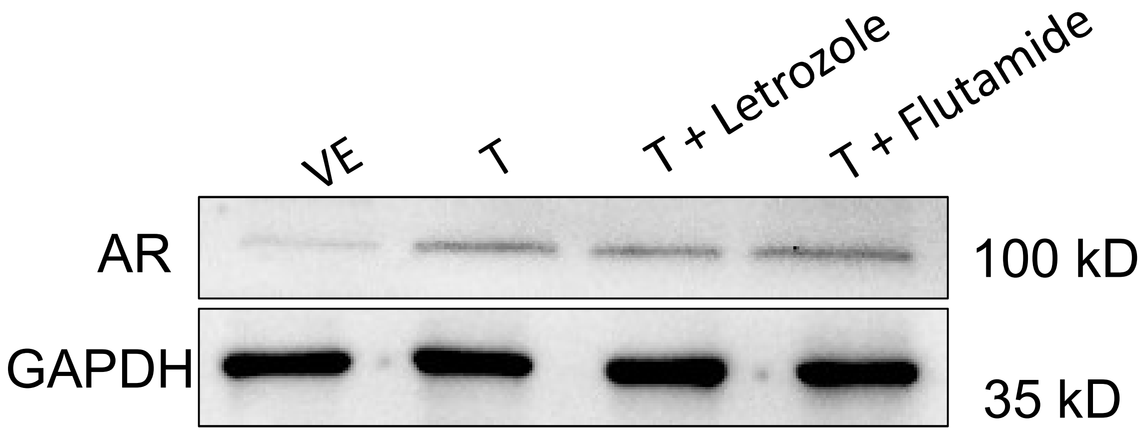
VE
T
T + Letrozole
T + Flutamide
AR
100 kD
GAPDH
35 kD

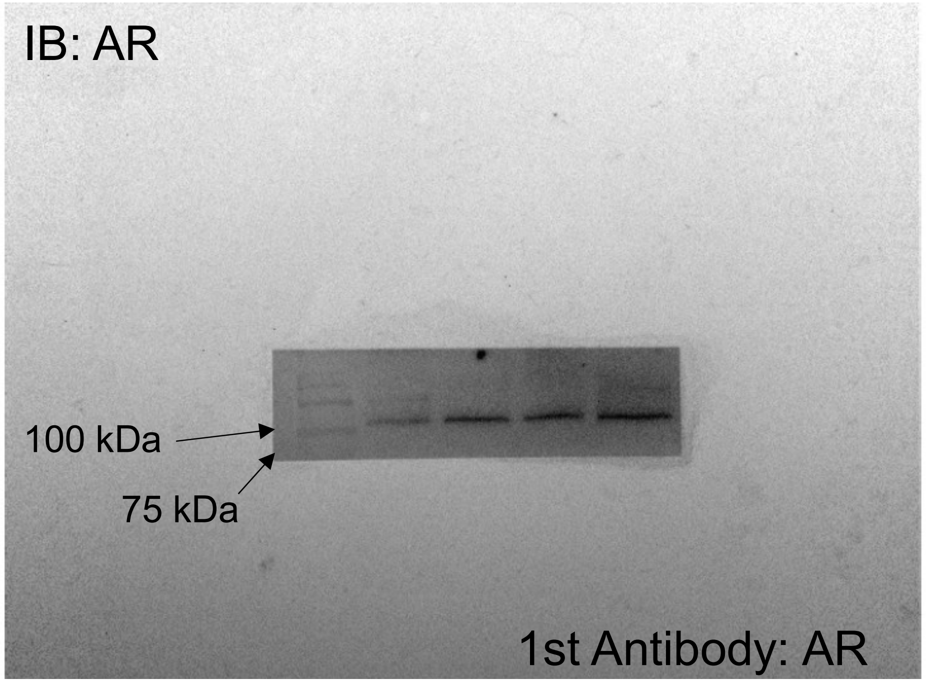
IB: AR
100 kDa
75 kDa
1st Antibody: AR

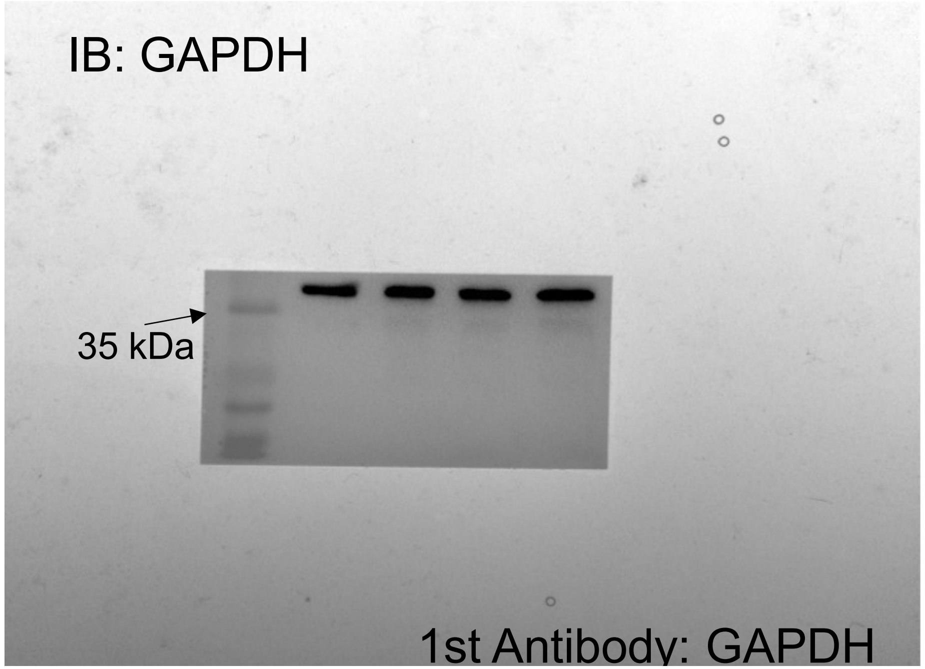
IB: GAPDH
35 kDa
1st Antibody: GAPDH
